# Investigating the Mechanism of Yiqi Huoxue Jieyu Granules Against Ischemic Stroke Through Network Pharmacology, Molecular Docking and Experimental Verification

**DOI:** 10.3390/ph18091332

**Published:** 2025-09-05

**Authors:** Ying Chen, Huifen Zhou, Ting Zhang, Haitong Wan

**Affiliations:** 1 Academy of TCM Cardio-Cerebrovascular Diseases, Zhejiang Chinese Medical University, Hangzhou 310053, China; chenying202403@126.com (Y.C.); zhouhuifen2320@126.com (H.Z.); 2 Zhejiang Key Laboratory of Chinese Medicine for Cardiovascular and Cerebrovascular Disease, Hangzhou 310053, China; 3 School of Life Science, Zhejiang Chinese Medical University, Hangzhou 310053, China; 4 School of Pharmaceutical Sciences, Zhejiang Chinese Medical University, Hangzhou 310053, China; 5 Academy of Chinese Medical Sciences, Henan University of Chinese Medicine, Zhengzhou 450046, China

**Keywords:** YHJG, network pharmacology, ischemic stroke (IS), inflammatory indexes, ERK

## Abstract

**Background:** Ischemic stroke (IS) is a significant cause of global mortality and disability. Yiqi Huoxue Jieyu granules (YHJGs) show therapeutic potential for IS, but their mechanisms remain unclear. This study investigated YHJGs’ effects through network pharmacology, molecular docking, and experimental validation. **Methods:** Active YHJG components and IS targets were identified from TCMSP, GeneCards, and DisGeNET databases. Network analysis and molecular docking (AutoDock Vina) were performed. In vivo studies used 72 male Sprague-Dawley rats (MCAO model) divided into sham, model, nimodipine (10.8 mg/kg), and three YHJG dose groups (0.72, 1.44, 2.88 g/kg). Assessments included neurological scores, TTC staining, histopathology, and molecular analyses (qPCR/Western blot). **Results:** Network analysis identified 256 shared targets between YHJG and IS, with PI3K-AKT and MAPK as key pathways. Molecular docking showed strong binding between YHJG compounds (e.g., quercetin) and core targets (AKT1, ERK1/2). YHJG treatment significantly improved neurological function (*p* < 0.01), reduced infarct volume (*p* < 0.01), and attenuated neuronal damage. The expression of IL-1β, TNF-α, IL-6, AKT1, and pERK1/2/ERK1/2 significantly increased in the MCAO group (*p* < 0.01), while YHJG treatment significantly reduced their expression (*p* < 0.01). PPAR-γ expression significantly increased in the YHJG-H group (*p* < 0.01). **Conclusions:** The expression of IL-1β, TNF-α, IL-6, AKT1, and pERK1/2/ERK1/2 significantly increased in the MCAO group, while YHJG treatment significantly reduced their expression. PPAR-γ expression significantly increased in the YHJG-H group. YHJGs could treat IS through diverse ingredients, targets, and pathways by inhibiting inflammatory indices and AKT1 expression, and reducing ERK1/2 phosphorylation.

## 1. Introduction

Globally, stroke represents one of the principal contributors to disability and mortality, ranking among the most frequent neurological diseases with an estimated lifetime risk between 8% and 10% [1]. As the third most common cause of death worldwide, it constitutes a significant public health challenge. Ischemic stroke (IS) and hemorrhagic stroke (HS) are the two primary categories, with IS responsible for over three-quarters of all cases [2]. The fundamental pathological mechanism underlying IS involves the formation of intravascular thrombi, leading to necrosis of cerebral tissues and subsequent focal neurological impairments [3]. At present, the main effective treatment is timely revascularization therapies, including intravenous thrombolysis and endovascular thrombectomy procedures in the early stage of ischemic stroke [4,5]. Current treatment methods still have some limitations. Thus, it is important to find a new replacement therapy for IS.

Traditional Chinese medicine (TCM) has unique advantages and rich experience in treating IS. YiqiHuoxue Jieyu Formula (YHJF) is composed of *Astragalus membranaceus* (Huang Qi), *Paeonia lactiflora* (Bai Shao), *Prunus persica* (Tao Ren), *Bupleurum chinense* (Chai Hu), *Ligusticum chuanxiong* (Chuan Xiong), *Curcuma aromatica* (Yu Jin), and *Acorus tatarinowii* (Shi Chang Pu) in a ratio of 2:1.67:1:0.67:1:1:0.67. YHJGs are formulated based on YHJF. YHJF is an effective formula for treating post-stroke depression, ischemic stroke (IS), the recovery period after a stroke, and the after-effects period (cerebral thrombosis, cerebral infarction). Previous studies have shown that Yiqi Hoxue Jieyu Decoction has an anti-PSD effect [6]. Also, it has been found to treat brain damage to some extent. In this paper, we attempt to validate its role in the treatment of ischemic stroke, combining network pharmacology and molecular docking.

The methodology of network pharmacology (NP) has emerged from the integration of pharmacological principles and systems biology, aiming to elucidate the potential mechanisms through which traditional Chinese medicine (TCM) exerts its therapeutic actions [7]. To elucidate the active constituents and underlying mechanisms of YHJGs, this study integrated network pharmacology (NP) with molecular docking techniques. Initially, bioactive compounds derived from YHJGs were identified and screened. Subsequently, the core targets associated with these components were systematically analyzed. A multi-level network illustrating compound-target interactions was constructed to explore the binding properties between phytochemicals and their corresponding targets. Finally, in vivo experiments were conducted to validate the computational predictions.

This study aimed to thoroughly evaluate the effect of YHJGs on neurogenesis in a rat model of MCAO. The main goal was to clarify how YHJGs impact inflammation, especially in the context of stroke-related brain injury. Additionally, the study sought to investigate the underlying mechanism.

## 2. Results

### 2.1. Active Compounds in YHJGs and Targets Prediction

A total of 97 active compounds of YHJGs were screened, including 20 from Huang Qi, 15 from Chai Hu, 7 from Chuan Xiong, 13 from Bai Shao, 15 from Yu Jin, 4 from Shi Chang Pu, and 23 from Tao Ren. We added six index ingredients, including Calycosin-7-glucoside, astragaloside IV, Saikosaponin D, Saikosaponin A, Ferulic Acid, and Amygdalin, excluding compounds that do not find the target. The total of 97 active compounds is shown in the Supplementary File S1.

### 2.2. Drug-Compound-Target Network Construction

We predicted the target through the Swiss Target Database and used UniProt to standardize the target name. After excluding duplicates, 768 targets were selected for the following research.

Using the Swiss Target Database and UniProt, targets were predicted and name-standardized. After removing duplicates, 768 unique targets were selected for further research.

**Figure 1:**
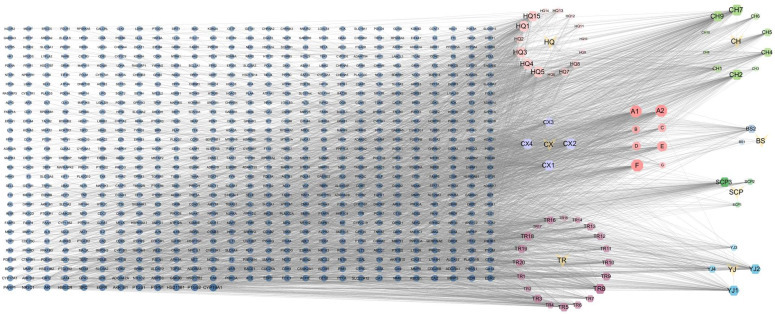
YHJG active ingredient-target network.

Cytoscape version 3.7.1 was used to visualize and analyze the interaction network between active ingredients of YHJGs and their targets. A comprehensive network comprising 841 nodes was generated, including 768 targets, 66 active ingredients, and seven drugs, along with a total of 3836 relationships. The nodes in the network graph were sized and shaded based on their degree values, with larger and darker nodes indicating higher degrees of connectivity. This reflects their substantial involvement in biological functions and signifies their importance within the biological context. The degree values of the active ingredients were analyzed using the software function network analysis (Figure 1). The top five ingredients are shown in Table 1.

### 2.3. Ischemic Stroke-Related Targets

After searching four databases, Genecards, OMIM, TTD, and Drug Bank, 1158, 20, 15, and 101 targets were obtained, respectively. After merging and removing duplicate targets, 1251 targets were finally obtained for the disease.

### 2.4. PPI Network Construction and Key Target Prediction

**Figure 2:**
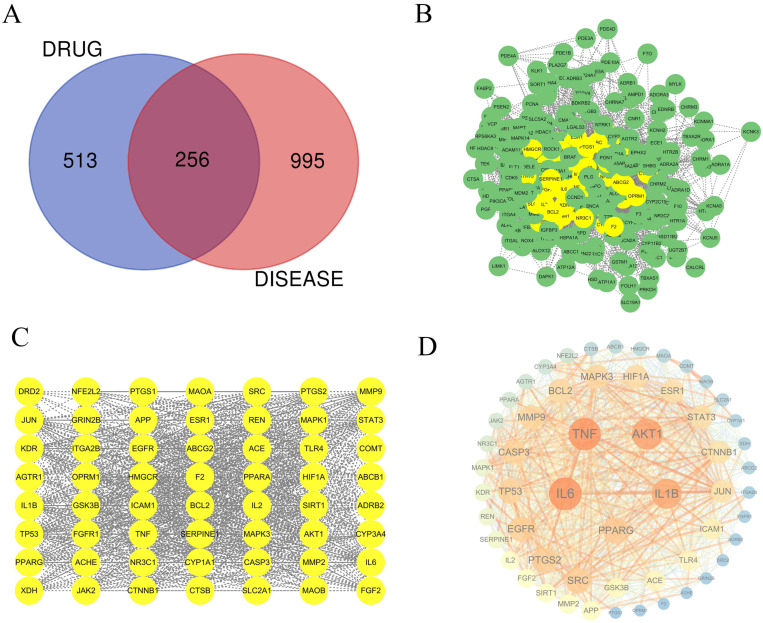
The method and the screening strategy for the key nodes of common targets. A Venn diagram image of common targets between YHJGs and IS, revealing a total of 256 common targets (**A**). 256 common targets were drawn into an image (**B**). Through a topological assessment, applying CentiScaPe, 56 key targets were screened (**C**). The key target nodes were sized and shaded based on their degree values in the network graph (**D**).

A Venn diagram analysis was conducted to identify the intersection of target genes between YHJGs and IS (Ischemic Stroke) target genes, revealing 256 common target genes (Figure 2A).

Furthermore, pivotal proteins related to YHJGs’ efficacy in treating IS were identified by constructing a PPI network using the STRING database. We imported data into the software Cytoscope3.7.1 (Figure 2B). Subsequently, three thresholds (DC > 37.75, BC > 257.687, and CC > 0.00198) were applied, which were calculated by CentiScaPe. This process resulted in the identification of 56 key target nodes (Figure 2C). The key target nodes in the graph were visually represented with a larger size and darker color to indicate higher degree values (Figure 2D), signifying their greater involvement in biological functions and importance in biology.

Among the key targets identified, the top five based on DC were IL6, TNF, AKT1, IL1B, and PPARG. These targets are particularly significant due to their extensive connectivity within the network, suggesting their pivotal roles in biological processes. The gene names of those gene symbols were *IL-6*, *TNF-α*, *AKT1*, *IL-1β*, and *PPAR-γ*.

### 2.5. GO and KEGG Pathway Enrichment Analysis

**Figure 3:**
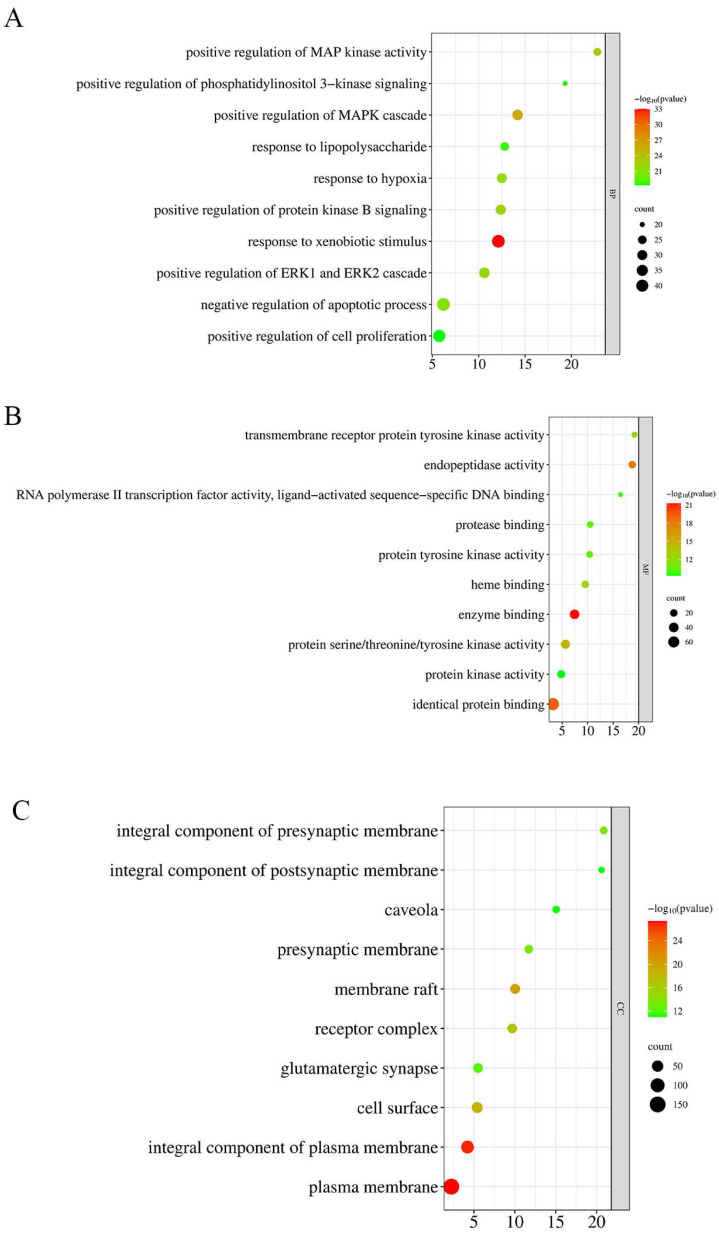

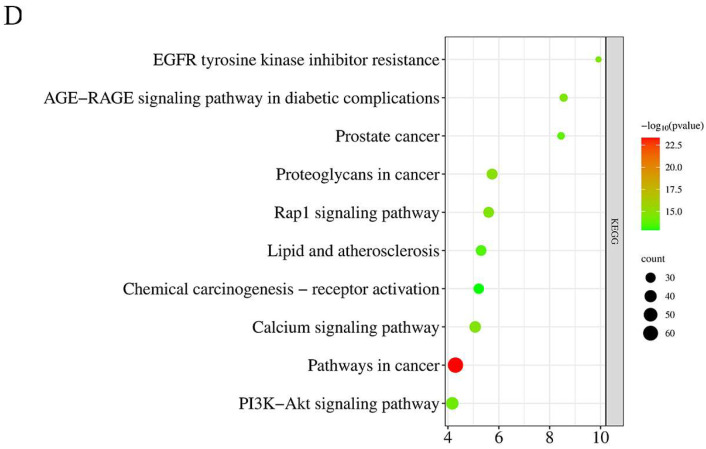
GO and KEGG enrichment analysis. Bubble chart of BP (**A**). Bubble chart of MF (**B**). Bubble chart of CC (**C**). Bubble chart of KEGG Analysis (**D**).

The official gene symbols of the intersection targets between YHJGs and IS were input into DAVID, with a significance threshold set at *p* ≤ 0.05 and FDR ≤ 0.05. Gene Ontology (GO) annotation and Kyoto Encyclopedia of Genes and Genomes (KEGG) pathway enrichment analyses were conducted, with the pre-analysis species set to “Homo sapiens”. Subsequently, bubble charts were generated using the Bioinformatics “www.bioinformatics.com.cn (accessed on 28 April 2024)” free online platform for bioinformatics-related data analysis, as illustrated in Figure 3.

The GO analysis includes three categories: biological process (BP), cellular component (CC), and molecular function (MF). BP is mainly concerned with response to xenobiotic stimuli, positive regulation of MAPK cascade, positive regulation of MAP kinase activity, positive regulation of protein kinase B signaling, positive regulation of ERK1 and ERK2 cascade, response to hypoxia, negative regulation of apoptotic process, response to lipopolysaccharide, and positive regulation of phosphatidylinositol 3-kinase signaling. CC is mainly concerned with the plasma membrane, cytoplasm, cytosol, integral component of membrane, nucleus, membrane, integral component of plasma membrane, nucleoplasm, and extracellular exosome. MF is mainly concerned with protein binding, identical protein binding, metal ion binding, ATP binding, enzyme binding, protein serine/threonine/tyrosine kinase activity, protein homodimerization activity, zinc ion binding, and protein kinase activity.

KEGG mainly concerns pathways in cancer, proteoglycans in cancer, EGFR tyrosine kinase inhibitor resistance, calcium signaling pathway, Rap1 signaling pathway, AGE-RAGE signaling pathway in diabetic complications, PI3K-Akt signaling pathway, prostate cancer, lipid and atherosclerosis, and chemical carcinogenesis–receptor activation.

### 2.6. Molecular Docking

**Figure 4:**
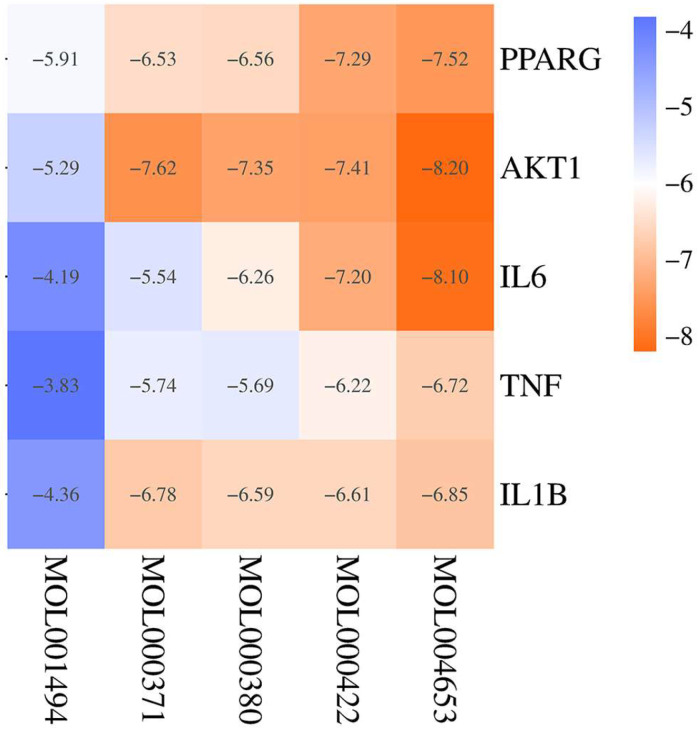
The cluster heatmap shows the difference in binding energy of molecular docking between the top 5 ingredients and the top 5 key targets.

The top five bioactive ingredients of YHJGs exhibited binding affinity with the top five core targets: *AKT1* (P31749), *IL-1B* (P01584), *IL6* (P05231), *TNF* (P01375), and *PPARG* (P37231). The Bioinformatics free online platform generated the heat map depicting the variance in binding energy between the top five ingredients and top five key targets. The legend was plotted at the right of the heatmap (Figure 4).

**Figure 5:**
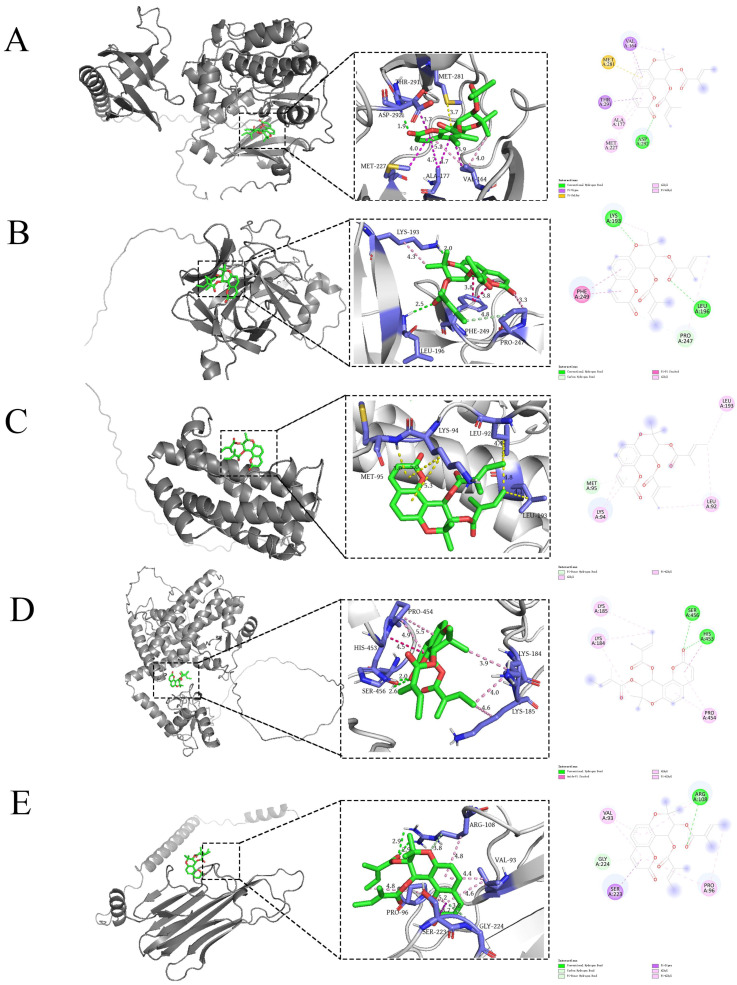
Binding sites of (+)-Anomalin and the top 5 key targets. The left of the image is a 3D global map of the binding site, the middle is a 3D local map of the binding site, and the right is a 2D map of the binding site: AKT1 (**A**), IL-1B (**B**), IL-6 (**C**), PPARG (**D**), TNF (**E**).

This suggested that YHJGs hold promise as a therapeutic approach for ischemic stroke through modulation of these key targets. The results showed that all the top five target proteins could strongly bind to the ingredient (+)-Anomalin. So, we focused on analyzing the binding sites of (+)-Anomalin with the target protein (Figure 5).

### 2.7. Experimental Verification

#### 2.7.1. HPLC Profile of YHJGs

**Figure 6:**
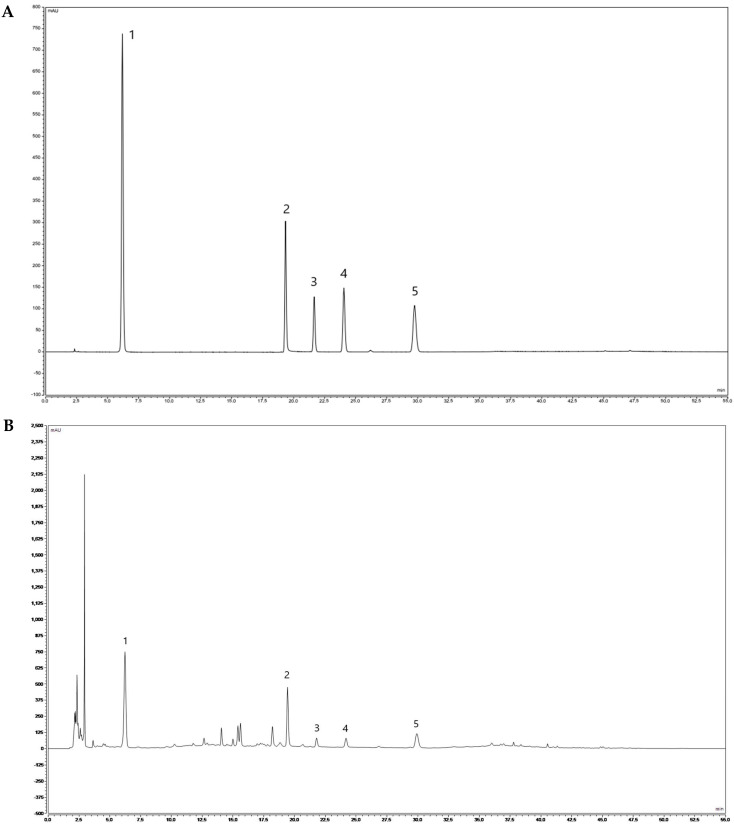
HPLC characterization of YHJGs. (**A**) Mixed reference standard solution, (**B**) Test solution of YHJG. 1 is gallic acid, 2 is paeoniflorin, 3 is Calycosin-7-glucoside, 4 is ferulic acid, and 5 is benzoic acid.

The qualitative determination of YHJGs was performed by HPLC. Five peaks were identified by comparing the retention times with the reference standard, and the main constituents of YHJG were as follows: gallic acid, paeoniflorin, Calycosin-7-glucoside, ferulic acid, and benzoic acid (Figure 6) (The detailed experiment is mentioned the Supplementary File S2).

#### 2.7.2. YHJGs Improved Neurological Deficits After MCAO in Rats

**Figure 7:**
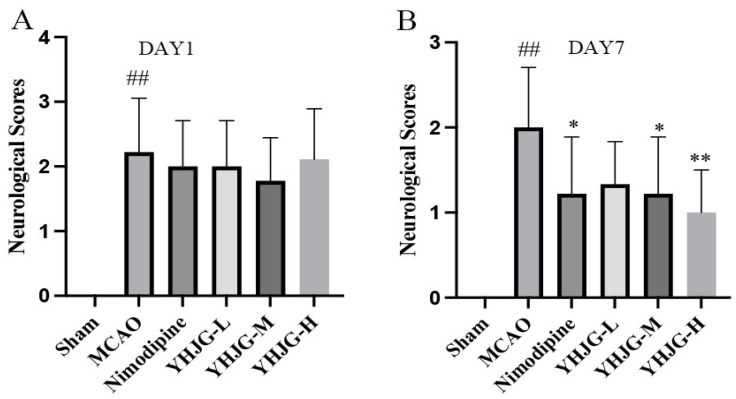
Rats’ neurological scores on day 1 and day 7. The neurological deficit scores of 1 day post-MCAO (**A**) and 7 days post-MCAO (**B**) were evaluated using the Longa method. (x ± SD, *n* = 9). ^##^ *p* < 0.01 vs. Sham group. * *p* < 0.05, ** *p* < 0.01 vs. MCAO group.

Before MCAO induction, the rats’ neurological function scores were 0, mirroring those of the sham group rats. On day 1 post-MCAO, there was no significant difference in neurological deficit scores between the MCAO group and the four treated groups (Figure 7A). However, by day 7, rats in the Nimodipine group and the YHJG-M group exhibited lower neurological scores compared to those in the MCAO group (*p* < 0.05), and rats in the YHJG-H group exhibited significantly lower neurological scores compared to those in the MCAO group (*p* < 0.01) (Figure 7B).

#### 2.7.3. YHJGs Reduced Infarction Volume and Infarction Rate After MCAO in Rats

**Figure 8:**
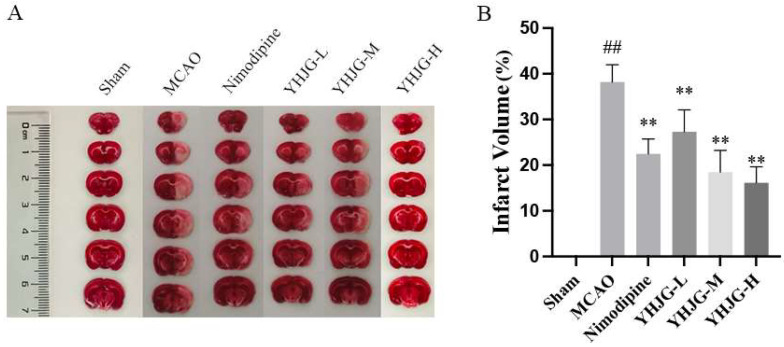
The representative images of TTC staining (**A**) and the infarct volume of the rats (**B**) (x ± SD, *n* = 6). ^##^ *p* < 0.01 vs. Sham group. ** *p* < 0.01 vs. MCAO group.

After 60 min of cerebral ischemia, reperfusion led to substantial neurological deficits and a notable cerebral infarction. As depicted in Figure 8A, a representative image of TTC staining illustrates the extent of infarction, while Figure 8B presents the infarct volume of the rats. In the Sham group, no infarction was observed in the rat brain. However, in the MCAO group, there was a significant increase in infarct volume compared to the Sham group (*p* < 0.01). Remarkably, the infarct volumes in the YHJG-treated groups and Nimodipine-treated group were significantly reduced compared to those in the MCAO group (*p* < 0.01).

**Figure 9:**
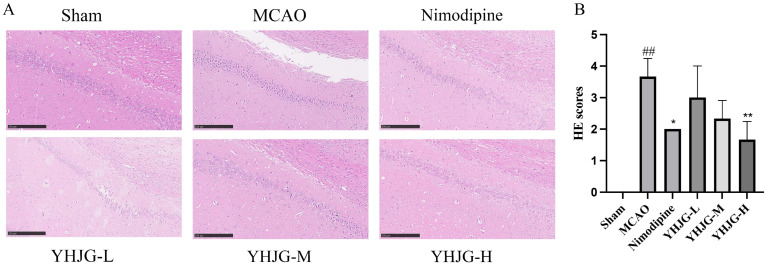
The representative images of H&E staining (**A**) and HE scores of the rats (**B**) (x ± SD, *n* = 3). ^##^ *p* < 0.01 vs. Sham group. * *p* < 0.05, ** *p* < 0.01 vs. MCAO group. Rats in the MCAO group displayed significant degeneration and necrosis of neurons in brain tissue, which represents the success of the MCAO model. The brain tissue morphology and structure of the YHJG-H group showed a significant improvement. Compared with the Sham group, the HE scores increased. On the contrary, HE scores in the nimodipine YHJG-H group decreased significantly compared with the MCAO group (Scale bar: 250 μm).

As shown in Figure 9, rats in the Sham group exhibited no evident pathological changes. In contrast, rats in the MCAO group displayed significant degeneration and necrosis of neurons in brain tissue, characterized by disordered cell arrangement and irregular morphology. However, compared to the MCAO group, the YHJG-treated groups and the nimodipine-treated group showed reduced brain tissue damage, with relatively regular cell arrangement and preserved cell morphology. Notably, rats in the YHJG-H group showed a significant improvement in brain tissue morphology and structure. Compared with the Sham group, the HE scores increased. On the contrary, HE scores in the nimodipine YHJG-H group decreased significantly compared with the MCAO group.

#### 2.7.4. YHJGs Alleviated the Damage to the Nissl Bodies in MCAO Rats

**Figure 10:**
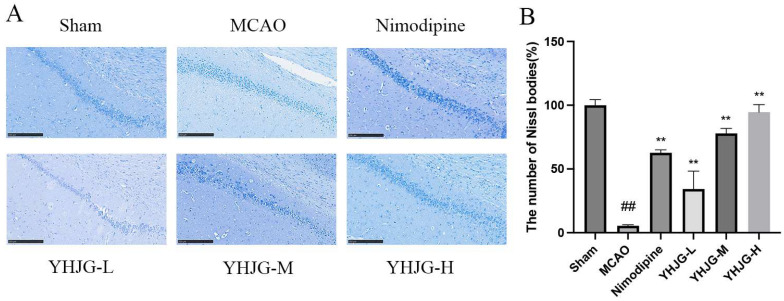
The representative images of Nissl staining (**A**) and the number of Nissl bodies (%) of the rats (**B**) (x ± SD, *n* = 3). ^##^ *p* < 0.01 vs. Sham group. ** *p* < 0.01 vs. MCAO group. Compared with the Sham group, the brain tissue of the MCAO group showed fewer Nissl bodies. Compared with the MCAO group, the brain tissue of the YHJG-treating and Nimodipine-treating groups showed more Nissl bodies. (Scale bar: 250 μm).

Results of Nissl staining are presented in Figure 10. In the Sham group, neurons were orderly arranged with clearly observable Nissl substance. Relative to the Sham group, the MCAO group showed a marked reduction in cytoplasmic Nissl bodies (*p* < 0.01), along with diminished staining intensity. Certain pyramidal cells in this group also displayed dissolved Nissl material, accompanied by indistinct cellular outlines and faint staining. By contrast, animals treated with YHJGs and Nimodipine exhibited notably restored cellular architecture and a significant rise in Nissl body density (*p* < 0.01) relative to the MCAO group.

#### 2.7.5. The Effects of YHJGs on the mRNA Levels of IL-1β, TNF-α, IL-6, AKT1, and PPAR-γ in the MCAO Rat Brain Tissue

**Figure 11:**
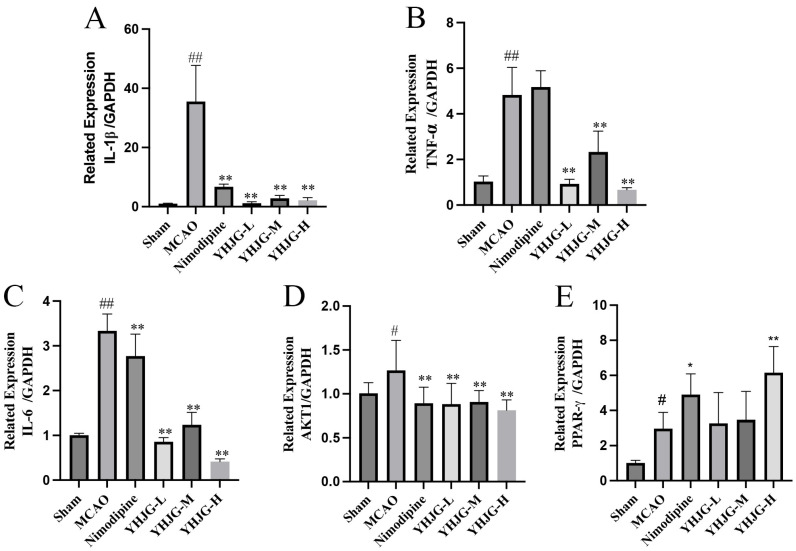
The mRNA levels of IL-1β (**A**), TNF-α (**B**), IL-6 (**C**), AKT1 (**D**), and PPAR-γ (**E**) in the brain tissue of rats (x ± SD, *n* = 3). ^#^ *p* < 0.05, ^##^ *p* < 0.01 vs. Sham group. * *p* < 0.05, ** *p* < 0.01 vs. MCAO group.

The mRNA levels of IL-1β, TNF-α, IL-6, AKT1, and PPAR-γ in rat brain tissues are depicted in Figure 11. Compared to the Sham group, the mRNA levels of IL-1β, TNF-α, IL-6, and AKT1 significantly increased in the MCAO group (*p*  <  0.01; *p*  <  0.05). Conversely, in the YHJG-treated groups, the mRNA levels of IL-6, IL-1β, TNF-α, and AKT1 were significantly reduced (*p*  <  0.01) compared to the MCAO group. Similarly, in the Nimodipine-treated group, the mRNA levels of IL-6, IL-1β, and AKT1 were significantly reduced (*p*  <  0.01) compared to the MCAO group. Additionally, compared to the Sham group, the mRNA levels of PPAR-γ significantly increased in the MCAO group (*p * <  0.05). Compared to the MCAO group, the mRNA levels of PPAR-γ significantly increased in the Nimodipine (*p * <  0.05) and YHJG-H (*p*  <  0.01) groups.

#### 2.7.6. YHJGs Down-Regulated the Expression of IL-1β, TNF-α, IL-6, AKT1, PPAR-γ, and pERK1/2/ERK 1/2 in the Ischemic Hemisphere After MCAO

**Figure 12:**
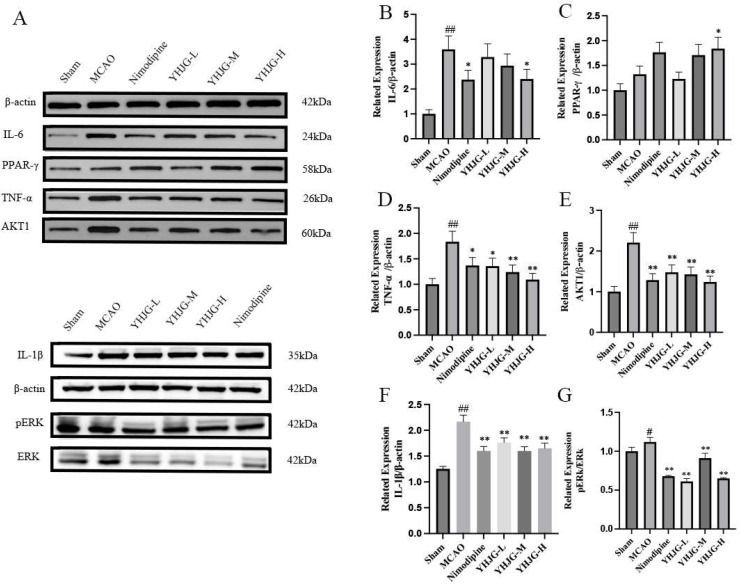
The representative images of western blotting (**A**); Using Western blotting to examine the related expression of IL-6/β-actin (**B**), PPAR-γ/β-actin (**C**), TNF-α/β-actin (**D**), AKT1/β-actin (**E**), L-1β/β-actin (**F**), and pERK/ERK (**G**) protein in rat (x ± SD, *n* = 3). ^#^ *p* < 0.05, ^##^ *p* < 0.01 vs. Sham group. * *p* < 0.05, ** *p* < 0.01 vs. MCAO group.

The expression of IL-1β, TNF-α, IL-6, AKT1, PPAR-γ, and pERK1/2/ ERK 1/2 in the rat brain tissues is shown in Figure 12. Compared to the Sham group, the expression of IL-1β, TNF-α, IL-6, AKT1, and pERK1/2/ERK 1/2 significantly increased in the MCAO group (*p*  <  0.01; *p*  <  0.05). Conversely, in the YHJG-treated groups, the expression of IL-1β, TNF-α, IL-6, AKT1, and pERK1/2/ERK 1/2 was significantly reduced (*p*  <  0.01; *p*  <  0.05) compared to the MCAO group. Similarly, in the Nimodipine-treated group, the expression of IL-1β, TNF-α, IL-6, AKT1, and pERK1/2/ERK 1/2 was significantly reduced (*p*  <  0.01; *p*  <  0.05) compared to the MCAO group. Additionally, compared to the MCAO group, the expression of PPAR-γ significantly increased in the YHJG-H group (*p * <  0.05).

## 3. Discussion

Among the selected active compounds, many studies have confirmed that they have a good therapeutic effect on ischemic stroke, such as Quercetin [8], Kaempferol [9] and so on. These active compounds may be the pharmacodynamic material basis of YHJGs in the treatment of IS.

In our research, the top five key targets were *IL6*, *TNF*, *AKT1*, *IL1β*, and *PPARG*. IL-6, IL1β, and TNF are common markers of inflammation. Most studies have mentioned the damage of inflammation after cerebral ischemia [10]. Inflammation can induce endothelial dysfunction leading to increased permeability of lipoproteins, subcutaneous accumulation, leukocyte recruitment, and platelet activation [11]. TNF-α is a multifunctional cytokine [12]. It plays a dual role in IS, triggering inflammation and neural damage while also potentially aiding in neuroprotection [13]. Existing studies have reported that AKT1 has therapeutic effects on stroke in terms of neuroprotection, promotion of neuronal regeneration and repair, anti-inflammatory effects, and vascular protection [14,15,16]. PPAR-γ also acts as a protective factor in combating the development of ischemic stroke [17,18].

The molecular docking results indicated that the top five target proteins could form stable bonds with the top five targets and exhibited strong binding affinity with the potential bioactive ingredient (+)-Anomalin. Anomalin is a seselin-type pyranocoumarin isolated for the first time from Angelica anomala Avé-Lal but is also found in several other plant species [19]. Anomalin can regulate the AP-1 signaling pathway and inhibit the production of pro-inflammatory mediators such as cytokines (IL-1β, IL-6, and TNF-α) [20].

The results of animal experiments showed that YHJG can improve neurological deficits, reduce infarction volume, and alleviate the damage to the Nissl bodies after MCAO in rats, which verified that YHJGs have a significant anti-IS effect.

YHJGs can reduce the mRNA levels of IL-1β, TNF-α, IL-6, and AKT1 in the MCAO rat brain tissue. Besides, YHJGs down-regulated the protein expression of IL-1β in the ischemic hemisphere after MCAO. AKT1 is pivotal in regulating the PI3K/Akt/mTOR pathway. Recent research indicates that inhibiting PI3K/Akt/mTOR signaling exacerbates neuronal apoptosis and autophagy in ischemic stroke [21,22]. The down-regulation of Akt1 can affect the NF-κB pathway, crucial in inflammation, reducing inflammatory factors and mitigating the response [23]. In addition, compared to the Sham group, the mRNA levels of PPAR-γ significantly increased in the brain tissue of rats in the MCAO group (*p*  <  0.05). The increase in PPAR-γ mRNA in the MCAO model group is likely a natural response to stroke-induced ischemic conditions, aiming to activate protective pathways. This helps reduce inflammation, enhance cell survival, and promote tissue repair to minimize brain damage [24]. Simultaneously, the significant upregulation observed in the YHJG-H compared to the MCAO model group suggests an enhanced effect due to drug intervention.

**Figure 13:**
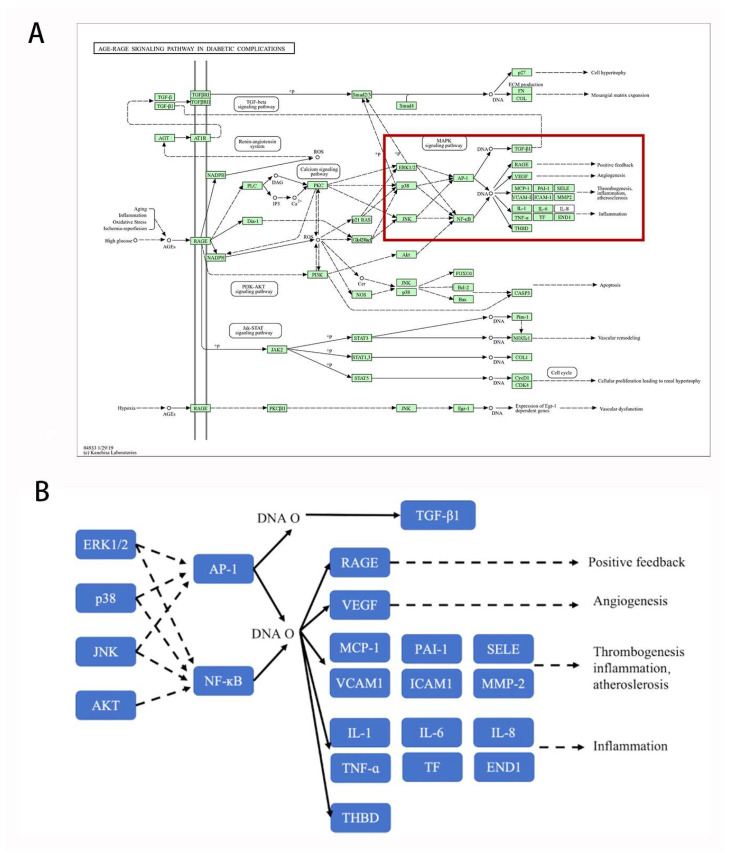
AGE-RAGE signaling pathway in diabetic complications. Signaling pathway provided by the DAVID database (**A**); Focused details of signaling pathway (**B**). Solid and dashed arrows denote specific and non-specific enzymatic reactions, respectively, as defined by the KEGG database.

YHJGs can reduce the inflammatory response related to cerebral ischemia-reperfusion injury. We found that part of the image of the AGE-RAGE signaling pathway in diabetic complications indicates the relation between protein kinase B signaling, MAPK cascade, ERK1 and ERK2 cascade, and pro-inflammatory mediators such as cytokines (IL-1β, IL-6, and TNF-α) (Figure 13). Mitogen-activated protein kinases (MAPKs) [25,26], including ERK1/2, p38, and JNK, as well as the PI3K/Akt signaling pathway, play crucial roles in regulating inflammatory responses by modulating key transcription factors such as AP-1 and NF-κB. Activation of these pathways leads to the transcription of pro-inflammatory cytokines like IL-1β, IL-6, and TNF-α, which contribute to inflammation. Conversely, inhibition of these pathways results in the downregulation of these cytokines, thus attenuating the inflammatory response.

As a result of our enrichment analysis, the Gene Ontology (GO) analysis revealed that YHJGs can have a positive regulation of the ERK1 and ERK2 cascade. Certain studies propose that ERK1/2 may act as neuroprotectants by suppressing oxidative stress, mitochondrial-dependent apoptosis [27], and neuronal cell death [28].

To further probe the mechanism, we performed Western blotting to examine the related expression of pERK1/2/ERK1/2. However, the result is the opposite of the results for GO analysis. We found that there was an apparent discrepancy between the GO analysis (predicting ERK1/2 pathway activation) and Western blot results (showing pERK1/2 downregulation at 7 days post-ischemia). This divergence can be reconciled through three interrelated explanations: First, the GO analysis reflects potential pathway modulation based on database-curated molecular interactions. Western blot quantifies the actual net effect of YHJGs’ multi-target regulation at our specific experimental endpoint. Second, in ischemic stroke models, ERK1/2 signaling is known to exhibit temporally distinct roles—early neuroprotective activation (<72 h) versus late pathological hyperactivity (7+ days) [29]. Our Western blot data at 7 days likely capture YHJGs’ therapeutic suppression of this maladaptive late-phase ERK activation, while the GO analysis encompasses both phases. Third, methodological differences exist as GO terms include all ERK-related processes (both positive and negative regulation), while our Western blot specifically measured phosphorylation status.

Our result shows that YHJGs inhibited the phosphorylation of ERK1/2. Studies also have suggested that inhibiting ERK1/2 may protect against inflammation, apoptosis, and blood–brain barrier (BBB) damage, indicating a potential benefit in reducing these effects [30]. Also, inhibition of ERK1/2 phosphorylation could have a neuroprotective effect on MCAO model rats [31]. These findings suggest that the signaling for Akt1 relevance and the ERK-dependent signal transduction changes during ischemic brain injury. During ischemic brain injury, YHJGs can decrease both AKT1 expression, potentially influencing the biological functions associated with the AKT1 signaling pathway, and the phosphorylation level of ERK1/2, thereby impacting the activity of the ERK1/2 signaling pathway and its related biological effects.

Based on our experimental results, YHJGs appear to effectively modulate inflammatory responses. YHJGs significantly inhibit the phosphorylation of ERK1/2 and decrease Akt1 protein levels, which may contribute to reduced pro-inflammatory cytokine expression. Studies have shown that astragalus polysaccharides alleviate LPS-induced inflammation via the NF-κB/MAPK signaling pathway [32]. AP-1 transcription factor JunD regulates ischemia/reperfusion brain damage via IL-1β [33]. Although the precise effects of YHJGs on AP-1 and NF-κB remain to be experimentally validated, the observed reduction in IL-1β, IL-6, and TNF-α suggests that YHJGs hold promise as a therapeutic agent for stroke-related inflammation.

However, network pharmacology has certain limitations that need to be considered: the accuracy of network pharmacology models is influenced by factors such as data quality, model selection, and parameter settings, leading to potential prediction errors or uncertainties. Some network pharmacology models can be highly complex and lack interpretability, making it challenging to understand the internal mechanisms and prediction outcomes, thereby limiting a deeper understanding of pharmacological issues. The intricate network of interactions in TCM formulations, where each component can interact with multiple targets and each target can be influenced by multiple components, poses significant challenges for network pharmacology analysis. This complexity makes interpreting and validating results more difficult. While network pharmacology offers significant potential and advantages, careful evaluation and effective strategies are needed to address these limitations in practical applications.

While this study provides preclinical evidence for the neuroprotective and anti-inflammatory effects of YHJGs, several key limitations must be addressed for successful clinical translation. First, animal models cannot fully replicate the complex etiology and comorbidities of human stroke. Second, the preventive administration strategy used in this experiment differs from the realistic therapeutic time window in clinical practice.

It is noteworthy that the parent formula of YHJGs, YHJF, has demonstrated efficacy in a clinical study on post-stroke depression (PSD) [6]. Given that PSD and ischemic stroke share key pathophysiological mechanisms such as neuroinflammation, this finding provides indirect support for the clinical potential of YHJGs. As a standardized granule formulation, YHJG offers improved bioavailability and dosing accuracy, which may further enhance its therapeutic effects.

Future translational research should focus on conducting Phase II clinical trials directly in acute ischemic stroke populations, with emphasis on: (1) determining the optimal therapeutic time window; (2) validating relevant biomarkers; and (3) evaluating compatibility with standard thrombolytic/antithrombotic therapies.

## 4. Materials and Methods

### 4.1. Data Collection and Processing

#### 4.1.1. Composite Ingredients of YHJGs

Data on bioactive constituents present in the seven herbal medicines comprising YHJGs were retrieved from the Traditional Chinese Medicine Systems Pharmacology (TCMSP) platform “https://old.tcmsp-e.com/tcmsp.php (accessed on 10 October 2023)”. [34].

#### 4.1.2. Screening of Bioactive Ingredients

TCM is primarily administered orally, leading us to screen the bioactive components of YHJGs based on two key parameters influencing gastrointestinal absorption: oral bioavailability (OB) ≥30% and drug-likeness (DL) ≥0.18. Combined with the previous research, related literature, and the Chinese Pharmacopoeia [35], six index ingredients were added, including Calycosin-7-glucoside (3′-Hydroxy-4′-methoxyisoflavone-7-*O*-beta-d-glucoside) [36] (from Huang Qi), astragaloside IV [37] (from Huang Qi), Saikosaponin D [38] (from Chai Hu), Saikosaponin A [39,40] (from Chai Hu), Ferulic Acid [41] (from Chuan Xiong), and Amygdalin [42] (from Tao Ren).

#### 4.1.3. Target Prediction of Bioactive Ingredients

Initially, we acquired the molecular structure of the bioactive compounds found in YHJGs along with their ‘canonical smiles’ and ‘sdf’ structural formulas from PubChem “https://pubchem.ncbi.nlm.nih.gov/ (accessed on 18 October 2023)”. Subsequently, we employed ‘Homo sapiens’ as the species to predict the targets using the Swiss Target Database. To standardize the target names and eliminate duplicates, we utilized the UniProt knowledge database “https://sparql.uniprot.org/ (accessed on 19 October 2023)”.

#### 4.1.4. Identification of Ischemic Stroke-Related Targets

We performed a search using the keyword ‘Ischemic stroke’ across multiple databases: Gene Cards “Gene, https://www.genecards.org/ (accessed on 19 October 2023)”, Online Mendelian Inheritance in Man “OMIM, https://omim.org/ (accessed on 6 November 2023)” Therapeutic Target Database “TTD, https://db.idrblab.net/ttd/ (accessed on 19 October 2023)”, and Drug Bank “https://www.drugbank.com/ (accessed on 19 October 2023)”. Subsequently, we compiled and summarized the findings from these four databases while eliminating any duplicate entries.

### 4.2. Network Construction

#### 4.2.1. Drug–Ingredient–Target Network Construction

We employed Cytoscape_v3.7.1 “http://www.cytoscape.org (accessed on 28 March 2024)”to construct the drug–ingredient–target network. Subsequently, we conducted a topological analysis using Network Analyzer to rank nodes based on their degree within the network.

#### 4.2.2. Obtaining Shared Targets Between YHJGs and Specific Diseases

To identify common targets between drug targets and disease targets, we utilized the “Draw Venn Diagram” online tool “http://bioinformatics.psb.ugent.be/webtools/Venn/ (accessed on 6 November 2023)”. This tool played a crucial role in generating Venn diagrams, visually illustrating the intersection between targets linked to YHJGs and targets associated with different diseases.

#### 4.2.3. Construction of PPI Network Diagram

The common gene data we obtained earlier were input into the STRING database “https://cn.string-db.org/ (accessed on 28 March 2024)”. Protein interaction data in this database are weighted and integrated, providing a reliability value. To construct protein–protein interaction (PPI) network diagrams, we specified ‘Homo sapiens’ as the species and exported the results as a “tsv” file. This file was then imported into Cytoscape_v3.7.1 to visualize the PPI network diagram.

#### 4.2.4. Key Target Prediction

Utilizing the CentiScape_v2.2 plugin “https://apps.cytoscape.org/apps/centiscape (accessed on 28 March 2024)” within Cytoscape, a topological assessment was conducted on the network to identify crucial targets. Developed by the Center for Biomedical Computing at the University of Verona, CentiScape computes specific centrality parameters that describe the network’s topology. [43]. The Key targets were screened by 3 main parameters: Degree centrality (DC), Betweenness centrality (BC), and Closeness centrality (CC).

### 4.3. GO and KEGG Pathway Enrichment Analysis

We conducted KEGG and GO enrichment analyses to predict potential prevention targets of phenolic acids in ischemic stroke prevention. The enrichment data were analyzed using the DAVID database, with a significance threshold set at *p* < 0.05 for both GO and KEGG predictions. We analyzed the results to further assess GO and KEGG pathway enrichment within the network, and we utilized Bioinformatics “www.bioinformatics.com.cn (accessed on 19 April 2024)”.

### 4.4. Molecular Docking

We obtained the structures of the top 5 target proteins from the Alphafold database [44] and saved them in PDB format. These structure files were then imported into the AutodockTool 1.5.6 software, where we added atomic charges and converted the files to pdbqt format after hydrogenation. The mol2 format file containing bioactive ingredients was imported into the AutodockTool 1.5.6 software for molecular docking analysis. Atomic charges were added to the file and saved in pdbqt format to prepare the ligands for docking simulations. Autodock Vina software 1.2.3 was employed to simulate molecular docking, assessing the binding affinity of the top 5 target proteins with the top 5 potential bioactive ingredients. The binding affinities served as the criteria for evaluation, with smaller binding affinities indicating better docking outcomes.

### 4.5. Experimental Verification

#### 4.5.1. Preparation of YHJGs

One gram of dry YHJGs is equivalent to 1.78 g of *Astragalus membranaceus* (Huang Qi), 3.34 g of *Paeonia lactiflora* (Bai Shao), 2 g of *Prunus persica* (Tao Ren), 1.34 g of *Bupleurum chinense* (Chai Hu), 2 g of *Ligusticum chuanxiong* (Chuan Xiong), 1.34 g of *Acorus tatarinowii* (Shi Chang Pu), and 2 g of *Curcuma aromatica* (Yu Jin). The drugs’ batch number and origin are shown in Table 2.

First, Shi Chang Pu, Chuan Xiong, and Yu Jin were soaked in 4 times the amount of water for 1 h and refluxed with boiling water for 6 h; then we collected the aromatic water, and filtrated it. Second, Huang Qi, Chai Hu, Tao Ren, and Bai Shao were added to the residue, and 6 times the amount of water was used as the solvent; it was refluxed with boiling water for 3 times, each time for 1 h. We combined the outcomes from step 1 and step 2. Finally, after the concentrated liquid dextrin was added, the powder was spray-dried, and β-cyclodextrin was added to the powder to prepare the granules.

Before administration, the granulated traditional Chinese medicine was thoroughly dissolved by stirring in boiling water. All prepared formulations were stored at 4 °C in a low-humidity environment.

These dosages of YHJF were based on previous studies. The daily clinical dose of YHJF was based on 72 g of raw herbs, with a yield of 22.2% when processed into YHJGs, resulting in 16 g. The dose used in rats by gavage was converted according to the human and animal equivalent dose conversion factors. Calculation formula:

Rat dose (mg/kg) = Human dose (mg/kg) × (Human Km/Rat Km)

Simplified rule:

Human weight ≈ 70 kg; 

Rat dose ≈ Human dose × 6.3.

Km is a pharmacokinetic conversion factor used to calculate the equivalent dose between species based on body surface area.

The calculated dose was 1.44 g/kg for the YHJG-M group, and 0.72 and 2.88 g/kg were specified for the YHJG-L and YHJG-H groups.

#### 4.5.2. HPLC Analysis

To verify the quality and stability of YHJGs, its principal bioactive constituents were characterized using high-performance liquid chromatography (HPLC). Reference standards for the preparation of control solutions were procured from the China Food and Drug Administration (Beijing, China). The Agilent ZORBAX Eclipse XDB-C18 column (250 mm × 4.6 mm, 5 μm) was used for analysis.

The mobile phase A was acetonitrile, mobile phase B was 0.1% phosphoric acid–water solution, the gradient elution was based on the reference object of Table 3, the detection wavelengths were 220 nm and 321 nm (switched at 23 min for 3 min), the flow rate was 1.0 mL/min, the column temperature was 25 °C, and the injection volume was 10 μL.

#### 4.5.3. Animals and Experimental Grouping

Male Sprague–Dawley (SD) rats (weighing 260–280 g) of specific pathogen-free (SPF) grade were utilized in this investigation. The animals were sourced from the Animal Experiment Center of Zhejiang Chinese Medical University and Shanghai SLAC Laboratory Animal Co., Ltd., Shanghai, China (Animal Qualification Certificate: SCXK Shanghai 2022–0004). All animal experiments were performed in compliance with the institutional guidelines for the care and use of laboratory animals established by the Animal Experimental Center of Zhejiang Chinese Medical University. The experimental protocol received formal approval from the Ethics Committee on Animal Research at the same institution (Approval Code: IACUC-20231023-19).

Focal cerebral ischemia in rats was induced via the middle cerebral artery occlusion (MCAO) method. Following anesthesia, animals were maintained at a constant rectal temperature of 37 °C using a thermostatically controlled heating pad. The surgical approach involved dissecting and exposing the right common carotid artery (CCA), internal carotid artery (ICA), and external carotid artery (ECA). A minimal incision was then created in the common carotid artery (CCA), through which a head-shaped nylon filament (CINONTECH, Beijing, China) was carefully introduced and advanced from the CCA into the internal carotid artery (ICA) approximately 18 mm to achieve occlusion of the middle cerebral artery. After 60 min of ischemia, the filament was withdrawn to restore blood flow, and the surgical site was sutured. Sham-operated rats underwent identical surgical dissection and vessel exposure without insertion of the filament or arterial occlusion.

Out of the 72 rats utilized in this study, 12 rats were allocated to the sham group, while 60 underwent MCAO induction. The 60 MCAO rats were randomly divided into five groups: the MCAO group, Nimodipine-treated group (10.8 mg/kg/day), low dose YHJG-treated group (YHJG-L, 0.72 g/kg/day), middle dose YHJG-treated group (YHJG-M, 1.44 g/kg/day), and high dose YHJG-treated group (YHJG-H, 2.88 g/kg/day). The drugs were given via gavage once daily for seven consecutive days at the specified time. During the same procedure, both the sham and MCAO groups received an equivalent volume of pure water.

#### 4.5.4. Determination of Neurological Defect Score

Each group of 9 rats was evaluated using the Longa method on day 1 and day 7. The scale ranged from 0 to 4. Specifically, 0 indicated no abnormalities (representing normal conditions), 1 indicated slight issues (such as failure to fully flex the left forepaw), 2 indicated moderate symptoms (like counterclockwise circling), 3 indicated severe symptoms (such as leaning to the left), and 4 indicated very serious deficits (such as unconsciousness, inability to walk independently, and lack of response to noxious stimuli).

#### 4.5.5. Measurement of Infarct Volume and Determination of Infarction Rate

The rats in each group were decapitated for TTC staining [45]. The fresh brains were immediately stored at −20 °C for 15 min after the removal of the cerebellum and then cut into 2 mm-thick coronal slices. The slices were placed in a 2% solution of 2,3,5-triphenyltetrazolium chloride (TTC) (Sigma, St. Louis, MO, USA) and incubated at 37 °C for 30 min. The stained slices were photographed, and the area of ischemic brain injury was calculated by a blinded observer using ImageJ software 1.53, expressed as the percentage of the infarct area. The infarction rate = (Sum of brain area of infarct volume)/(Sum of brain area of each section) ×100%.

#### 4.5.6. Hematoxylin and Eosin (H&E) Staining Was Used to Observe the Pathological Changes in the Brain Tissue

Following decapitation, brain tissues from each group of rats were embedded in paraffin. The resulting sections were subjected to deparaffinization and sequentially hydrated through a graded ethanol series, followed by rinses in distilled water. Hematoxylin and eosin (H&E) staining was performed, after which the samples were dehydrated in xylene and mounted with neutral balsam. Neural histopathological alterations were examined under a microscope. Representative images were captured, and regions of interest within the samples were subsequently analyzed.

The scoring was based on the morphological structure of brain cells and the surrounding conditions: 0 points for normal cells; 1 point for a small amount of cell damage with no vacuoles around the cells; 2 points for obvious cell damage with a small number of vacuoles around the cells; 3 points for obvious cell damage with numerous vacuoles around the cells; and 4 points for extensive cell damage with a large number of vacuoles around the cells [46].

#### 4.5.7. Nissl Staining Was Used to Examine Neuronal Damage in the Rat Brain Tissue

First, the sections were deparaffinized and washed three times with water. Subsequently, they were immersed in a nylon staining solution at 60 °C for 20 min, followed by another three water washes and drying at 60 °C. After being rendered transparent with xylene, the sections were sealed with neutral gum. Neuronal damage in the rat brain tissue was then observed under a microscope. The Nissl bodies in each slice were independently counted by two individuals in a double-blind manner, and the average count was recorded for further analysis [47].

#### 4.5.8. Quantitative RT-PCR Was Used to Examine Gene Expression

Total RNA extraction was carried out using the FastPure Cell/Tissue Total RNA Isolation Kit V2 from Vazyme (Nanjing, Jiangsu, China), following the manufacturer’s instructions. Subsequently, the RNA concentration was determined. Then, reverse transcription was performed. Quantitative polymerase chain reaction was performed using the SYBR premix kit (ABclonal, Wuhan, Hubei, China). The well-mixed samples were added to the wells of the 96-well plate, and the PCR reaction was carried out under the following conditions: 95 °C, 3 min, denaturation; 95 °C, 5 s; 60 °C, 30 s, 40 cycles. The primer sequence (Sangon Biotech, Shanghai, China) list is shown in Table 4. All RT-PCR was performed at least 3 times.

#### 4.5.9. Western Blotting Was Used to Examine Protein Expression of IL-1β, TNF-α, IL-6, AKT1, PPAR-γ and pERK1/2/ ERK 1/2

Brain tissue protein content was extracted using a cold lysis buffer solution, and the protein concentration was assessed utilizing the BCA kit (Beyotime, Shanghai, China). Subsequently, SDS-PAGE electrophoresis was performed, followed by transferring the gel onto a PVDF membrane. It was Blocked with 5% skimmed milk powder for an hour, and then incubated with the primary antibody (Mouse anti *IL1β*, 1:1000, Mouse anti *β-actin*, 1:1000, Mouse anti *pERK*, 1:1000, Mouse anti *ERK*, 1:1000, all of our antibody was bought from Proteintech, Wuhan, Hubei, China) (Rabbit anti *IL-6*, 1:1000, Rabbit anti *TNF-α*, 1:1000, Rabbit anti *AKT*, 1:1000, Rabbit anti PPAR-γ, 1:1000, all of our antibody was bought from Servicebio, Wuhan, Hubei, China) at 4 °C on a shaker for 24 h. Following this, secondary antibody (HRP-conjugated goat-anti-mouse, 1:1000, Proteintech, Wuhan, Hubei, China; HRP-conjugated goat-anti-rabbit,1:3000, Servicebio, Wuhan, Hubei, China) was applied to the PVDF membrane, which was then incubated on the shaker. Finally, an ECL chemiluminescence instrument was utilized to visualize the membrane.

#### 4.5.10. Statistical Analysis

All numerical data were expressed as mean ± standard Deviation (SD). One-way analysis of variance (ANOVA) was conducted using GraphPad Prism 6.0. Statistical significance was considered when *p* < 0.05.

## 5. Conclusions

The therapeutic effect of YHJGs on IS is attributed to their action through multiple ingredients, targets, and pathways. Through our initial investigation, we unveiled the effective ingredients and molecular mechanisms underlying YHJGs’ efficacy in treating IS. Molecular docking studies, complemented by experimental validation, provided preliminary confirmation. The results provided strong evidence that treatment with YHJGs significantly improved neurological deficit symptoms, reduced stroke volume, mitigated pathological damage, and decreased levels of inflammatory factors.

In summary, our study highlights YHJGs’ potential as a novel treatment approach for managing inflammation associated with stroke. However, further research is needed to validate its effects on AP-1 and NF-κB and to assess its long-term efficacy and safety in clinical settings. Future studies should focus on elucidating the exact molecular mechanisms of YHJGs and evaluating their therapeutic potential in clinical trials to optimize their role in inflammatory disease management.

## Figures and Tables

**Table 1:** Top 5 ingredients.

MOLDID	Label	Drug	Component Name
MOL004653	CH7	Chai Hu	(+)-Anomalin
MOL001494	CX2	Chuan Xiong	Mandenol
MOL000371	HQ3	Huang Qi	3,9-di-*O*-Methylnissolin
MOL000380	HQ5	Huang Qi	(6a*R*,11a*R*)-9,10-Dimethoxy-6a,11a-dihydro-6*H*-benzofurano[3,2-*c*]chromen-3-ol
MOL000422	F	Huang Qi, Chai Hu, Bai Shao, and Shi Chang Pu	kaempferol

**Table 2:** The drugs’ batch number and origin.

Drug Name	Chinese Name	Batch Number	Manufacture Company	Place of Origin
*Astragalus membranaceus*	Huang Qi	201222	Jiaxing Oriental Chinese Medicine Decoction Pieces Co., Ltd., Jiaxing, Zhejiang, China	Neimenggu, China
*Ligusticum chuanxiong*	Chuan Xiong	201211		Sichuan, China
*Bupleurum chinense*	Chai Hu	201215		Hebei, China
*Prunus persica*	Tao Ren	201123		Shandong, China
*Acorus tatarinowii*	Shi Chang Pu	200826		Zhejiang, China
*Paeonia lactiflora*	Bai Shao	201212		Anhui, China
*Curcuma aromatica*	Yu Jin	201201	Zhejiang University of Chinese Medicine Decoction Pieces Co., Ltd., Hangzhou, Zhejiang, China	Zhejiang, China

**Table 3:** Chromatographic gradient elution table.

Time (min)	Acetonitrile (A%)	0.1% Phosphoric Acid–Water (B%)
0	5	95
5	5	95
10	13	87
16	18	82
23	19.7	80.3
26	19.7	80.3
32	25	75
45	55	45
55	5	95

**Table 4:** Primer sequence list.

Gene	Forward Primer	Reverse Primer
*TNF-α*	CTAGAGACAGCCGCATCTTCTTG	GTAGTTGAGGTCAATGAAGGGGT
*IL-1β*	GGCAGGTCTACTTTGGAGTCATT	CTCCACGGGCAAGACATAGG
*IL-6*	AGGCTGACAGACCCCAAAAG	TAGCCACTCCTTCTGTGACTCTA
*AKT1*	CGACGTAGCCATTGTGAAGGAG	ATTGTGCCACTGAGAAGTTGTTG
*PPAR-γ*	TGAATCCAGAGTCCGCTGACC	CGCCCTCGCCTTTGCTTTG
*GAPDH*	GACATGCCGCCTGGAGAAAC	AGCCCAGGATGCCCTTTAGT

## Data Availability

Data presented in this study is contained within the article and Supplementary Material. Further inquiries can be directed to the corresponding authors.

## Supplementary Materials

The following supporting information can be downloaded at: https://www.mdpi.com/article/10.3390/ph18091332/s1, File S1: YHJG Active compounds table. File S2: HPLC results supplementary.
