# Supplementary material for: Investigating the Mechanism of Yiqi Huoxue Jieyu Granules Against Ischemic Stroke Through Network Pharmacology, Molecular Docking and Experimental Verification
Source: Pharmaceuticals (Basel). 2025 Sep 5;18(9):1332. doi: 10.3390/ph18091332 (PMC12472206; doi:10.3390/ph18091332)
Supplement: Supplementary file 1 [file pharmaceuticals-18-01332-s001.zip › Supplementary File S1. YHJG Active Compounds.pdf]

Supplementary Table YHJG Active Compounds

Drug	MOLDID	Label	Active compound Name
**Huang Qi**	MOL000239	HQ1	Jaranol
	MOL000033	HQ2	(3S,8S,9S,10R,13R,14S,17R)-10,13-dimethyl-17-[(2R,5S)-5-propan-2-yloctan-2-yl]-2,3,4,7,8,9,11,12,14,15,16,17-dodecahydro-1H-cyclopenta[a]phenanthren-3-ol
	MOL000371	HQ3	3,9-di-O-methylnissolin
	MOL000378	HQ4	7-O-methylisomucronulatol
	MOL000380	HQ5	(6aR,11aR)-9,10-dimethoxy-6a,11a-dihydro-6H-benzofurano[3,2-c] chromen-3-ol
	MOL000387	HQ6	Bifendate
	MOL000392	HQ7	formononetin
	MOL000417	HQ8	Calycosin
	MOL000439	HQ9	isomucronulatol-7,2'-di-O-glucosiole
	MOL000442	HQ10	1,7-Dihydroxy-3,9-dimethoxy pterocarpene
	MOL000379	HQ11	9,10-dimethoxypterocarpan-3-O-β-D-glucoside
	MOL000418	HQ12	3'-Hydroxy-4'-methoxyisoflavone-7-O-beta-D-glucoside
	MOL000407	HQ13	astragalosideIV
	MOL000374	HQ14	5'-hydroxyiso-muronulatol-2',5'-di-O-glucoside
	MOL000438	HQ15	(3R)-3-(2-hydroxy-3,4-dimethoxyphenyl) chroman-7-ol
**Chai Hu**	MOL004628	CH1	Octalupine
	MOL004598	CH2	3,5,6,7-tetramethoxy-2-(3,4,5-trimethoxyphenyl) chromone
	MOL002776	CH3	Baicalin
	MOL001645	CH4	Linoleyl acetate
	MOL004718	CH5	α-spinasterol
	MOL000449	CH6	Stigmasterol
	MOL004653	CH7	(+)-Anomalin
	MOL004635	CH8	saikosaponin a
	MOL004609	CH9	Areapillin
	MOL004637	CH10	Saikosaponin D
**Chuan Xiong**	MOL002135	CX1	Myricanone
	MOL001494	CX2	Mandenol
	MOL000360	CX3	FER
	MOL002151	CX4	senkyunone
**Bai Shao**	MOL001919	BS1	(3S,5R,8R,9R,10S,14S)-3,17-dihydroxy-4,4,8,10,14-pentamethyl-2,3,5,6,7,9-hexahydro-1H-cyclopenta[a]phenanthrene-15,16-dione
	MOL001924	BS2	paeoniflorin
**Yu Jin**	MOL004328	YJ1	naringenin
	MOL004263	YJ2	(E)-5-Hydroxy-7-(4-hydroxyphenyl)-1-phenyl-1-heptene
	MOL004291	YJ3	Oxycurcumenol
	MOL004305	YJ4	Zedoalactone A
**Shi Chang Pu**	MOL003576	SCP1	(1R,3aS,4R,6aS)-1,4-bis(3,4-dimethoxyphenyl)-1,3,3a,4,6,6a-hexahydrofuro[4,3-c] furan
	MOL003578	SCP2	Cycloartenol
	MOL003542	SCP3	8-Isopentenyl-kaempferol
**Tao Ren**	MOL001351	TR1	Gibberellin A44
	MOL001353	TR2	GA60
	MOL001344	TR3	GA122-isolactone
	MOL001349	TR4	4a-formyl-7alpha-hydroxy-1-methyl-8-methylidene-4aalpha,4bbeta-gibbane-1alpha,10beta-dicarboxylic acid
	MOL001329	TR5	2,3-didehydro GA77
	MOL001360	TR6	GA77
	MOL001339	TR7	GA119
	MOL001340	TR8	GA120

	MOL001342	TR9	GA121-isolactone
	MOL001355	TR10	GA63
	MOL001361	TR11	GA87
	MOL001352	TR12	GA54
	MOL001328	TR13	2,3-didehydro GA70
	MOL001368	TR14	3-O-p-coumaroylquinic acid
	MOL000493	TR15	campesterol
	MOL001358	TR16	gibberellin 7
	MOL001320	TR16	Amygdalin
	MOL001348	TR17	gibberellin 17
	MOL001343	TR18	GA122
	MOL001350	TR19	GA30
	MOL001323	TR20	Sitosterol alpha1
**Common in Huang Qi and Chai Hu**	MOL000354	A1	isorhamnetin
	MOL000098	A2	quercetin
**Common in Chuan Xiong and Bai Shao**	MOL000359	B	sitosterol
**Common in Bai Shao, Yu Jin and Tao Ren**	MOL000358	C	beta-sitosterol
**Common in Huang Qi and Bai Shao**	MOL000211	D	Mairin
**Common in Huang Qi andTao Ren**	MOL000296	E	hederagenin
**Common in Huang Qi, Chai Hu, Bai Shao and Shi Chang Pu**	MOL000422	F	kaempferol
**Common in Huang Qi and Chuan Xiong**	MOL000433	G	FA
