# Supplementary material for: Investigating the Mechanism of Yiqi Huoxue Jieyu Granules Against Ischemic Stroke Through Network Pharmacology, Molecular Docking and Experimental Verification
Source: Pharmaceuticals (Basel). 2025 Sep 5;18(9):1332. doi: 10.3390/ph18091332 (PMC12472206; doi:10.3390/ph18091332)
Supplement: Supplementary file 1 [file pharmaceuticals-18-01332-s001.zip › Supplementary File S2. HPLC results Supplementary.pdf]

HPLC characterization of Yiqi Huoxue Jieyu granules(YHJG)

Yiqi Huoxue Jieyu granule is a kind of granule made by clinical experience and modern pharmaceutical technology of traditional Chinese medicine granule, which is made by extraction, concentration, spray drying, tablet and granulation etc. In order to better control the quality of particles, HPLC was used to establish the characteristic spectrum of Yiqi Huoxue Jieyu granule and improve the quality standard of particles, so as to provide reference for particle quality control.

1.Materials and reagents

Table S1 Main reagents and materials

Name	Batch Number	Manufacture Company
Gallic acid	110831-201906	China Institute for Food and Drug Control
Calycosin-7-glucoside	111920-201907	China Institute for Food and Drug Control
Paeoniflorin	110736-202044	China Institute for Food and Drug Control
Benzoic acid	100419-201703	China Institute for Food and Drug Control
Ferulic acid	110773-201012	China Institute for Food and Drug Control
Chromatographic grade methanol	L1263607304	Kommanditgesellschaft auf Aktien - KGaA
Chromatographic grade acetonitrile	JB128130	Kommanditgesellschaft auf Aktien - KGaA
Chromatograde phosphoric acid	P112025	Shanghai Aladdin Biochemical Technology Co., LTD
YHJG	211101	Huisong Pharmaceuticals Co. Ltd
YHJG	211102	Huisong Pharmaceuticals Co. Ltd
YHJG	211103	Huisong Pharmaceuticals Co. Ltd
YHJG	20231019	Zhejiang Chinese Medical University self-made
YHJG	20231021	Zhejiang Chinese Medical University self-made
YHJG	20231024	Zhejiang Chinese Medical University self-made
YHJG	20231026	Zhejiang Chinese Medical University self-made
YHJG	20231028	Zhejiang Chinese Medical University self-made
YHJG	20231029	Zhejiang Chinese Medical University self-made
YHJG	20231101	Zhejiang Chinese Medical University self-made
Lack of *Paeoniae Radix Alba*-negative granules	221019	Zhejiang Chinese Medical University self-made
Lack of *Astragalus membranaceus*-negative granules	221203	Zhejiang Chinese Medical University self-made
Lack of *chuanxiong Rhizom*-negative granules	231019	Zhejiang Chinese Medical University self-made

2.Method

1.Chromatographic Condition

The chromatographic column selected for this experiment was: Agilent model SB-C18 column (parameters: 4.6 mm × 250 mm, 5 μm), the mobile phase A was acetonitrile and mobile phase B was 0.1% phosphoric acid, the gradient elution was based on the reference object of Table S2, and the detection wavelengths were: 220 nm, 321 nm (switched at 23 min for 3 min), the flow rate was 1.0 mL/min, the column temperature was 25 °C, and the injection volume was 10 μL.

Table S2 Chromatographic gradient elution table

Time（min）	acetonitrile（%）	0.1% phosphoric acid（%）
0	5	95
5	5	95
10	13	87
16	18	82
23	19.7	80.3
26	19.7	80.3
32	25	75
45	55	45
55	5	95

2.Solution Preparation

2.1 Preparation of control solution

Appropriate amount of gallic acid, paeoniflorin, paeoniflorin, mullein isoflavone glucoside, ferulic acid and benzoic acid control was weighed precisely, and the mixture of the control solution was diluted with 50% methanol to the mass concentration of 128 μg/mL, 321 μg/mL, 33 μg/mL, 32 μg/mL and 33 μg/mL, respectively.

2.2 Preparation of test solution

2.2.1 Prepare the blank solution

Measure 25 mL ethanol into 100 mL volumetric bottle, add pure water to the scale, and obtain 25% ethanol solution.

2.2.1 Prepare the test product solution

Appropriate amount of the test article was finely ground, weighed precisely 0.5 g, and put inside a conical flask. Then add 10 mL of 25% ethanol, weigh and put it into the ultrasonic apparatus for ultrasonic treatment (power set at 300 W, frequency 40 kHz, time duration 30 minutes). After following the above steps, the sample was weighed and the weight loss was calculated, and then 25% ethanol was added to make up for the weight loss. After sufficient shaking, the supernatant was centrifuged and filtered through 0.22 μm microporous membrane, and the filtrate was taken as the filtrate.

2.3 Prepare a negative sample solution

Take appropriate amount of negative particles without *Astragalus membranaceus*, without *chuanxiong Rhizom* and without *Paeoniae Radix Alba* respectively and grind them finely, weigh 0.5 g precisely, and put them inside a conical flask. Add 10 mL of 25% ethanol, and put into the ultrasonic apparatus for ultrasonic treatment (power set at 300W, frequency 40 kHz, time duration 30 minutes), and continue to weigh according to the above steps, and after calculating the weight lost, add 25% ethanol solution to make up for the lost weight. After sufficient shaking, the supernatant was centrifuged and filtered through 0.22 μm microporous filter membrane, and the filtrate was taken as the filtrate.

3.Methodological Examination

3.1 Specialized examinatio

Take the appropriate amount of blank solution, negative sample solution, control solution, test solution, under the conditions of "1" in accordance with the experimental requirements of the sample determination.

3.2 Precision test

Precisely take up the test solution of "2.2", and inject the sample continuously for 6 times under the chromatographic conditions of "1", and then take the second peak of Paeonia lactiflora as the reference object, import the data into the software, and then calculate and compare the relative retention time of the common peaks and the RSD value of the relative peak area. The relative retention time of each common peak and the RSD value of the relative peak area were calculated and compared.

3.3 Repeatability test

Weigh a certain amount of particles in accordance with the experimental requirements, the specific operation of the method in accordance with the "2.2", and then make 6 copies of the test solution in parallel to meet the requirements of the chromatographic conditions in the "1" into the sample, the system software automatically record the chromatogram, determine the retention time of the experiment, and record the peak area integral value, specifically the second peak paeoniflorin peak as a reference object to calculate the relative retention time and relative peak area RSD value of each common peak. The system software automatically recorded the chromatograms, determined the retention time of the experiment, and recorded the peak area integral value, specifically with the second peak paeoniflorin peak as the reference object, calculated the relative retention time of the peaks and the relative peak area of the RSD value.

3.4 Stability test

Prepare the test solution according to the operation requirements of item “2.2”, and then inject the sample under the chromatographic conditions of item “1” at room temperature at 0, 2, 4, 6, 8, 10, 12 and 24 h. Determine the retention time and the integrated value of the peak area, and then calculate the relative retention time and RSD value of each common peak, taking the second peak of paeoniflorin as the reference. The RSD values of relative retention time and relative peak area of each common peak were calculated using the second peak of paeoniflorin as reference.

4.Establishment of characteristic map

Take 10 batches of granules, prepare 10 batches of test solution according to item “2.2”, inject the sample under the conditions of item “1” for detection, and import the chromatographic information obtained into the “Evaluation system of similarity of chromatographic profiles of traditional Chinese medicine” (2012 version) for systematic analysis. The chromatographic information obtained was imported into the “Evaluation System of Similarity of Chinese Medicine Chromatographic Profiles” (2012 version) for systematic analysis, and the selected reference chromatogram was the chromatogram of sample S1, using the median method, with the width of the time window set at 0.1 min, and the multi-point correction was performed to match the marker peaks, and to establish a superimposed chromatogram and a control chromatogram.

5.Similarity evaluation

The similarity of the feature profiles of 10 batches was calculated using the “Chinese medicine chromatographic fingerprint similarity evaluation system” (2012 version), in which the profile of the first sample (S1) was used as the reference profile of the feature profiles.

3.Result

1.Methodological Examination

1.1 Specialized examinatio

1 is gallic acid, 2 is paeoniflorin, 3 is Calycosin-7-glucoside, 4 is ferulic acid, and 5 is benzoic acid. In each exclusive negative solution, no corresponding peaks appeared at the retention time positions corresponding to the indicator components in the test solution. It indicates that the method has good specificity. The results are shown in Figure S1.

A

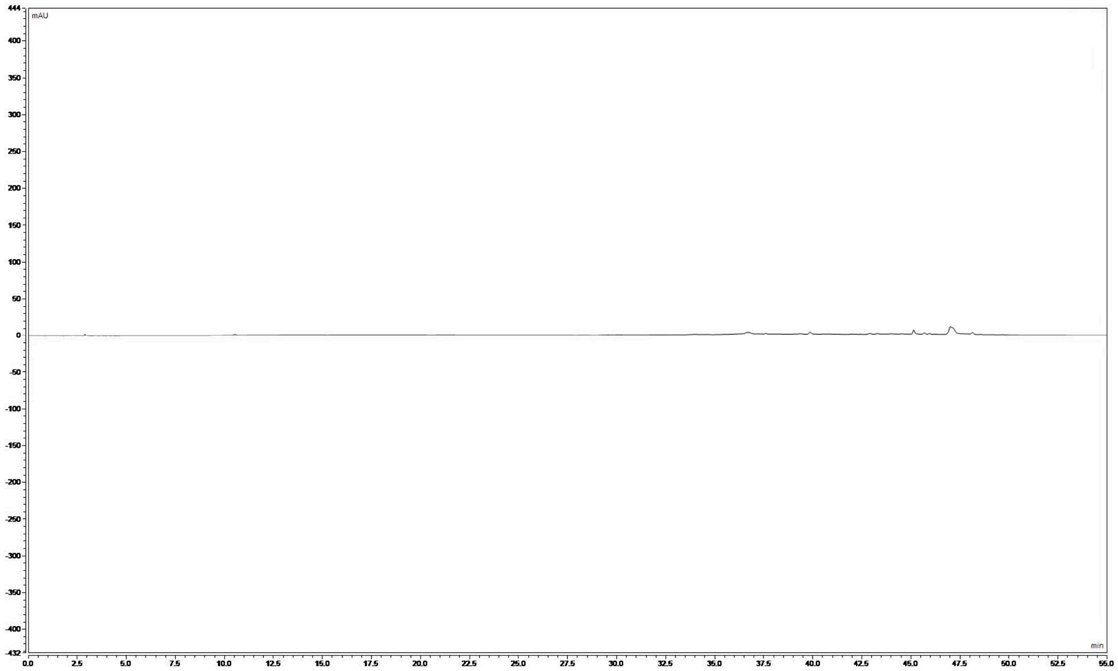

B

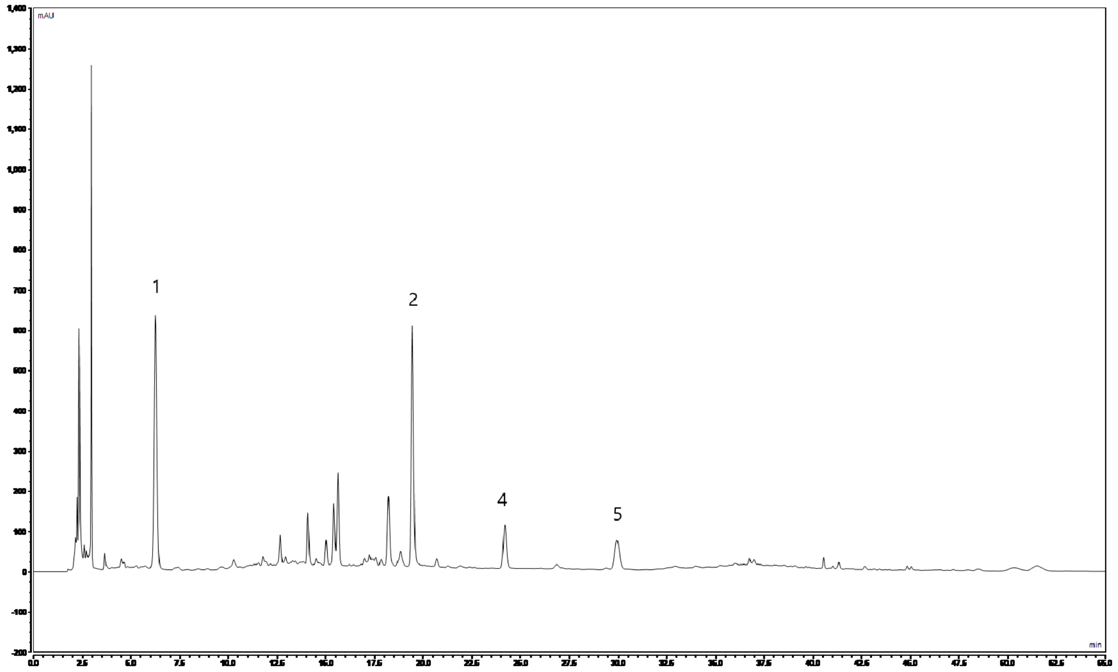

C

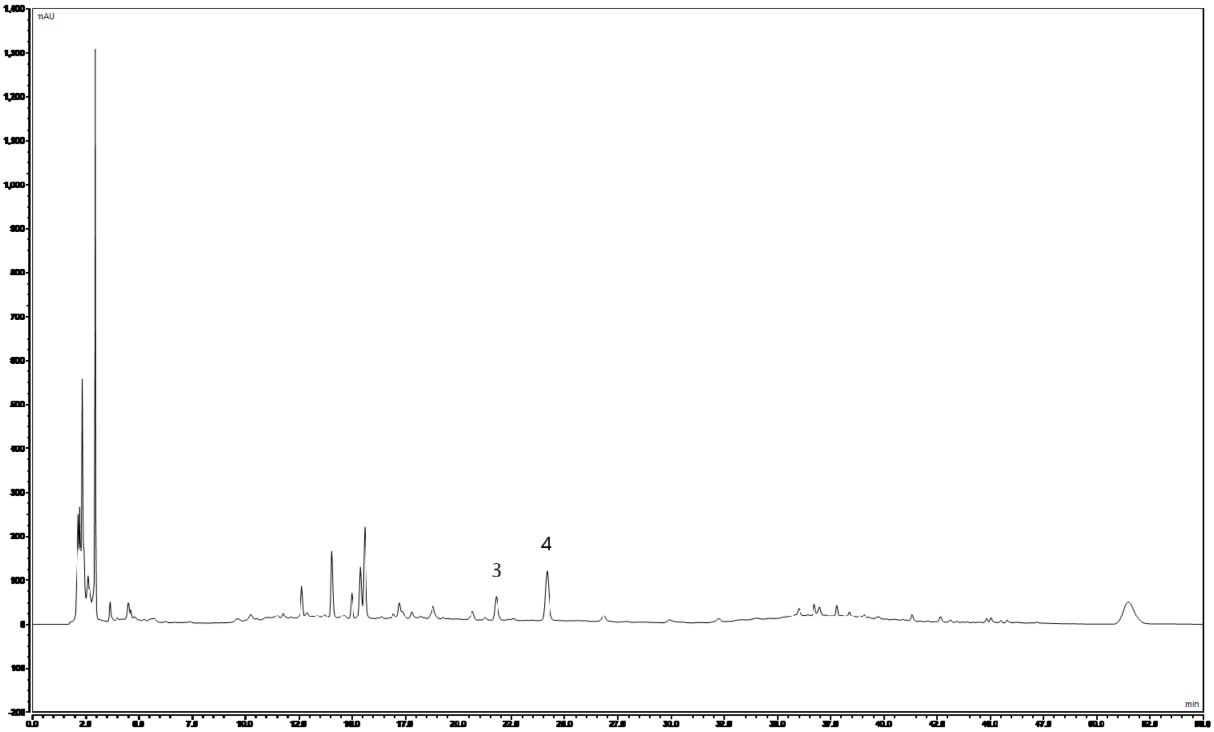

D

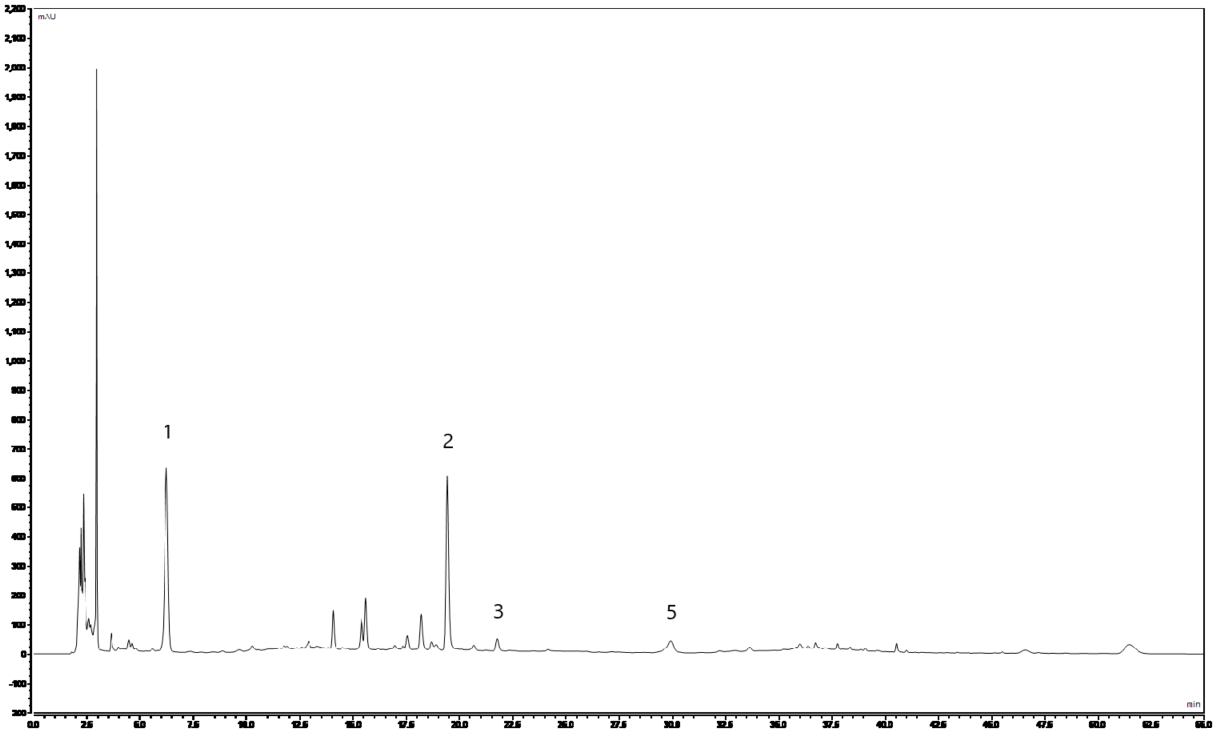

E

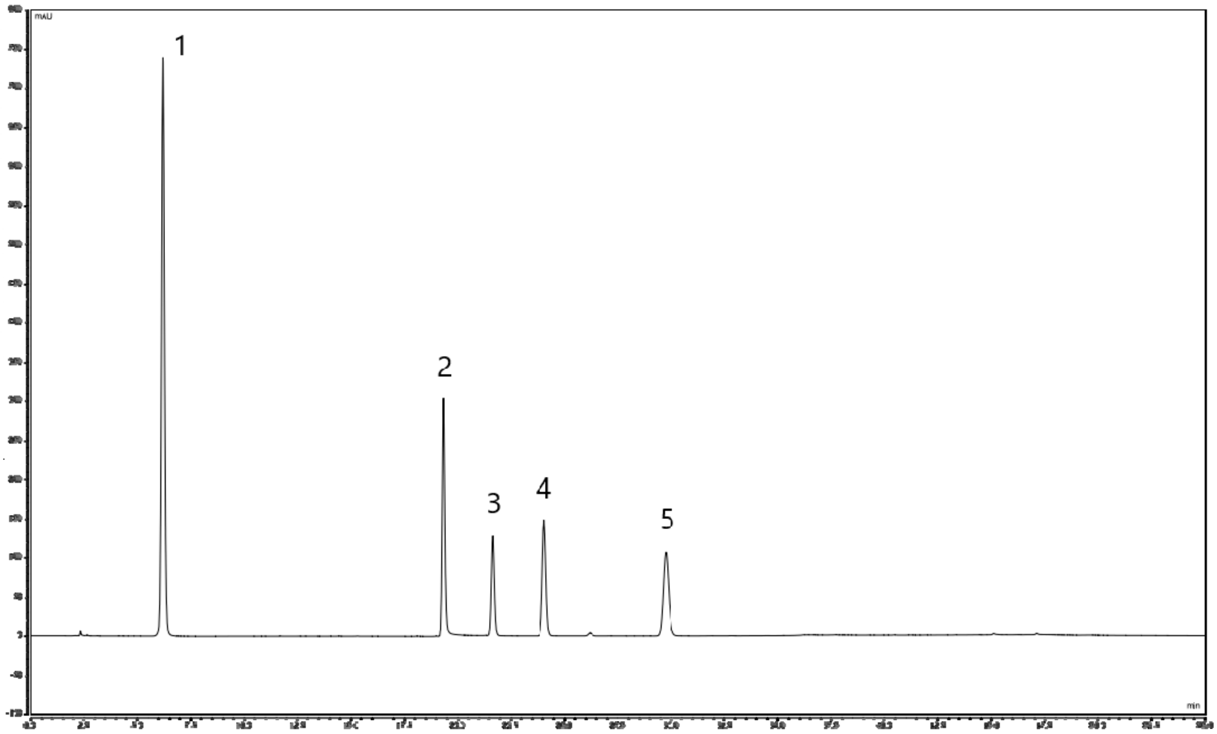

F

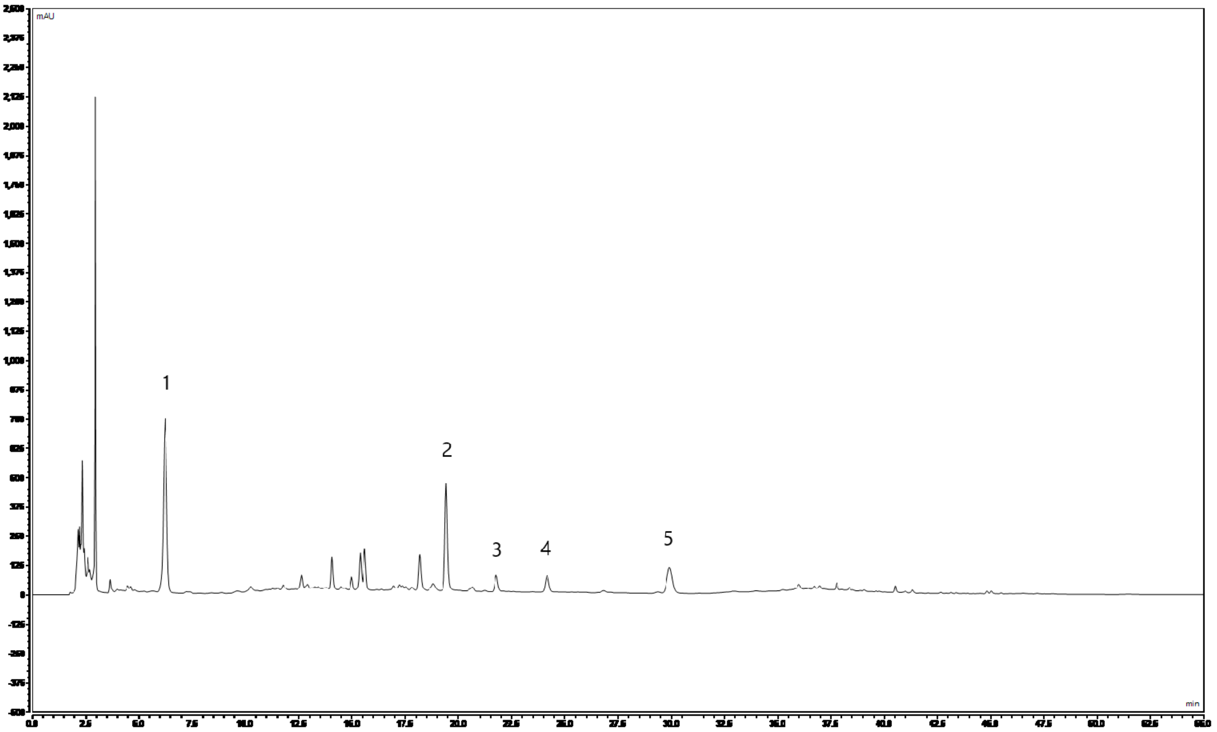

Figure S1 Specialized chromatograms, A. Blank solution; B. Deficient Astragalus negative sample solution; C. Deficient Paeonia lactiflora negative sample solution; D. Deficient Ligusticum chuanxiong negative sample solution; E. Mixed control solution; F. Test solution

1.2 Precision test

The results are shown in Table S3-1 and Table S3-2. The relative retention time is less than 0.72% and the relative peak area is less than 1.20%, which shows that the instrument has good precision and meets the experimental requirements.

Table S3-1 Precision test relative retention time

Number	1	2	3	4	5	6	RSD/%
1	0.111	0.110	0.111	0.111	0.111	0.111	0.071
2	0.325	0.325	0.324	0.324	0.324	0.324	0.032
3	0.724	0.724	0.724	0.724	0.724	0.724	0.014
4	0.804	0.804	0.804	0.804	0.804	0.804	0.010
5	0.937	0.937	0.937	0.937	0.937	0.937	0.007
6	0.969	0.969	0.969	0.969	0.969	0.969	0.004
7	1.000	1.000	1.000	1.000	1.000	1.000	0.000
8	1.122	1.122	1.121	1.121	1.121	1.121	0.012
9	1.246	1.246	1.246	1.246	1.246	1.246	0.005
10	1.543	1.543	1.542	1.543	1.543	1.543	0.042

Table S3-2 Precision test relative peak area

Number	1	2	3	4	5	6	RSD/%
1	0.312	0.311	0.310	0.303	0.305	0.311	1.199
2	2.183	2.178	2.173	2.170	2.171	2.174	0.219
3	0.220	0.221	0.221	0.221	0.221	0.221	0.145
4	0.348	0.347	0.348	0.348	0.349	0.348	0.168
5	0.327	0.326	0.325	0.325	0.326	0.327	0.260
6	0.114	0.113	0.113	0.113	0.113	0.113	0.344
7	1.000	1.000	1.000	1.000	1.000	1.000	0.000
8	0.182	0.182	0.182	0.182	0.181	0.182	0.159
9	0.228	0.228	0.228	0.228	0.228	0.228	0.079
10	0.512	0.512	0.513	0.513	0.513	0.514	0.093

1.3 Repeatability test

The results are shown in Table S4-1 and Table S4-2. The relative retention time is less than 1.41% and the relative peak area is less than 2.79%, indicating that the method has good repeatability.

Table S4-1 Repeatability test relative retention time

Number	1	2	3	4	5	6	RSD/%
1	0.111	0.111	0.111	0.111	0.111	0.111	0.081
2	0.323	0.323	0.323	0.323	0.323	0.323	0.055
3	0.724	0.724	0.724	0.724	0.724	0.724	0.009
4	0.793	0.793	0.793	0.793	0.793	0.793	0.009
5	0.887	0.887	0.887	0.887	0.887	0.887	0.016
6	0.937	0.969	0.937	0.937	0.937	0.937	1.405
7	1.000	1.000	1.000	1.000	1.000	1.000	0.000
8	1.122	1.122	1.122	1.122	1.121	1.122	0.006
9	1.244	1.244	1.244	1.244	1.244	1.244	0.008
10	1.541	1.541	1.541	1.541	1.541	1.541	0.010

Table S4-2 Repeatability test relative peak area

Number	1	2	3	4	5	6	RSD/%
1	0.157	0.150	0.157	0.156	0.150	0.150	2.421
2	0.999	0.990	0.983	0.989	0.991	0.991	0.504
3	0.199	0.199	0.199	0.199	0.199	0.199	0.130
4	0.369	0.369	0.352	0.348	0.350	0.367	2.782
5	0.067	0.068	0.068	0.069	0.068	0.069	0.820
6	0.244	0.245	0.245	0.251	0.246	0.246	1.064
7	1.000	1.000	1.000	1.000	1.000	1.000	0.000
8	0.158	0.159	0.158	0.162	0.159	0.159	0.870
9	0.233	0.232	0.232	0.231	0.232	0.232	0.259
10	0.526	0.523	0.524	0.522	0.524	0.524	0.257

1.4 Tability test

The results are shown in Table S5-1 and Table S5-2. The relative retention time is less than 1.05% and the relative peak area is less than 1.26%, indicating that the tested solution remains stable within 24 hours.

Table S5-1 Relative retention time of stability test

Time/h	0	2	4	6	8	10	12	24	RSD/%
1	0.111	0.111	0.111	0.111	0.111	0.111	0.111	0.111	0.089
2	0.320	0.325	0.324	0.325	0.324	0.324	0.325	0.316	1.045
3	0.721	0.722	0.722	0.722	0.722	0.722	0.722	0.719	0.139
4	0.803	0.804	0.804	0.804	0.804	0.804	0.804	0.801	0.102
5	0.936	0.937	0.937	0.937	0.937	0.937	0.937	0.936	0.040
6	0.969	0.968	0.968	0.968	0.968	0.968	0.968	0.969	0.033
7	1.000	1.000	1.000	1.000	1.000	1.000	1.000	1.000	0.000
8	1.121	1.121	1.121	1.121	1.121	1.121	1.121	1.122	0.026
9	1.110	1.110	1.110	1.110	1.110	1.110	1.110	1.109	0.029
10	1.541	1.540	1.541	1.540	1.540	1.540	1.541	1.539	0.027

Table S5-2 Stability test relative peak area

Time/h	0	2	4	6	8	10	12	24	RSD/%
1	0.265	0.268	0.265	0.265	0.265	0.266	0.269	0.268	0.651
2	2.204	2.199	2.195	2.194	2.190	2.185	2.182	2.167	0.529
3	0.217	0.217	0.218	0.218	0.217	0.218	0.217	0.211	1.035
4	0.349	0.347	0.347	0.347	0.346	0.348	0.346	0.335	1.253
5	0.328	0.326	0.327	0.325	0.326	0.326	0.325	0.326	0.261
6	0.110	0.112	0.111	0.112	0.111	0.111	0.111	0.109	0.869
7	1.000	1.000	1.000	1.000	1.000	1.000	1.000	1.000	0.000
8	0.180	0.181	0.180	0.180	0.180	0.180	0.181	0.180	0.156
9	0.228	0.228	0.228	0.232	0.228	0.227	0.228	0.227	0.704
10	0.513	0.515	0.513	0.516	0.514	0.515	0.514	0.501	0.904

2.Establishment of characteristic map

The chromatograms of YHJG were analyzed using the software of "Chinese medicine chromatographic fingerprint similarity evaluation system" (2012 version). 10 batches of YHJG had a total of 10 peaks, and the HPLC superimposed fingerprints and the control spectrum are shown in Figure S2.

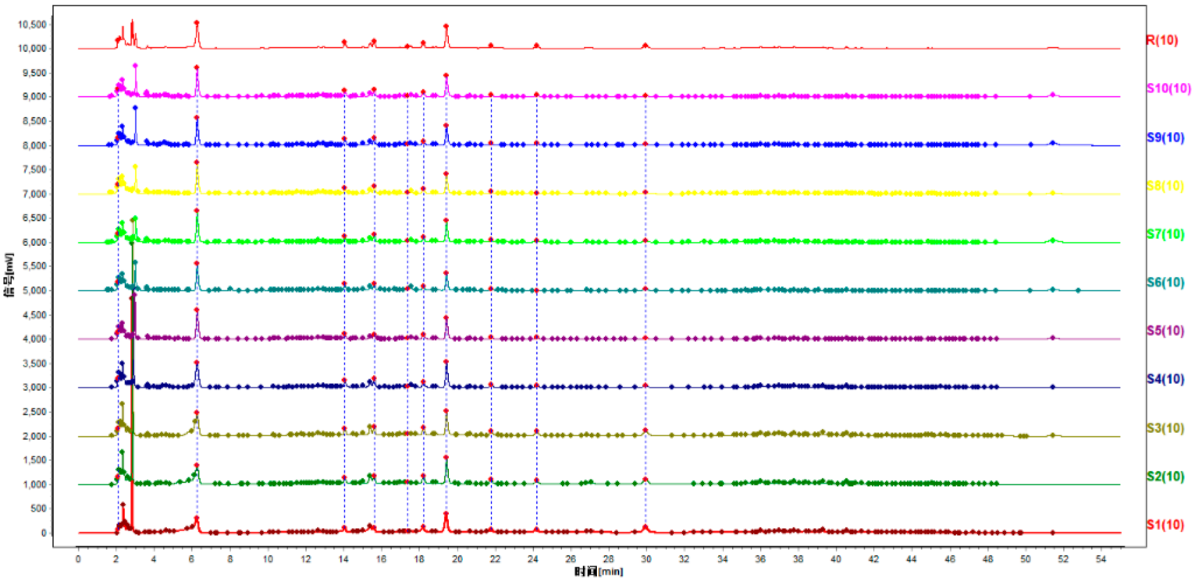

Figure S2 Combination Atlas of Yiqi Huoxue Jieyu Granules in 10 Batches (S1-S10) and Control Atlas (R)

3.Similarity evaluation

The similarity of the characteristic maps of 10 batches was calculated, in which the maps of the sample of batch 1 (S1) were used as the reference maps for the characteristic maps, as shown in Table S6. The similarity between the characteristic maps of 10 batches of YHJG and the control maps was greater than 0.90, which indicated that the chemical compositions of YHJG of different batches were basically the same, and the quality was stable.

Table S6 Results of similarity evaluation of characteristic map of 10 batches of Yiqi Huoxue Jieyu formula granules

Batches	S1	S2	S3	S4	S5	S6	S7	S8	S9	S10
S1	1.000	0.982	0.992	0.933	0.910	0.912	0.921	0.907	0.920	0.927
S2	0.982	1.000	0.993	0.973	0.956	0.965	0.965	0.960	0.967	0.970
S3	0.992	0.993	1.000	0.951	0.929	0.939	0.943	0.934	0.945	0.950
S4	0.933	0.973	0.951	1.000	0.994	0.994	0.998	0.994	0.996	0.998
S5	0.910	0.956	0.929	0.994	1.000	0.986	0.990	0.99	0.996	0.989
S6	0.912	0.965	0.939	0.994	0.986	1.000	0.996	0.999	0.992	0.996
S7	0.921	0.965	0.943	0.998	0.990	0.996	1.000	0.997	0.994	0.999
S8	0.907	0.960	0.934	0.994	0.990	0.999	0.997	1.000	0.994	0.995
S9	0.920	0.967	0.945	0.996	0.996	0.992	0.994	0.994	1.000	0.995
S10	0.927	0.970	0.950	0.998	0.989	0.996	0.999	0.995	0.995	1.000
R	0.956	0.988	0.973	0.996	0.987	0.991	0.994	0.99	0.993	0.995

3.Conclusions

In conclusion, the HPLC profiles of YHJG granules were established in this experiment and analyzed for several batches of granules, and 10 common peaks were found, with similarity greater than 0.90, and five components were recognized, namely gallic acid, paeoniflorin, Calycosin-7-glucoside, ferulic acid and benzoic acid, and the established method was stable and reproducible, which can provide a basis and reference for the quality control of YHJG granules.
